# SARS-CoV-2 proteases PLpro and 3CLpro cleave IRF3 and critical modulators of inflammatory pathways (NLRP12 and TAB1): implications for disease presentation across species

**DOI:** 10.1080/22221751.2020.1870414

**Published:** 2021-01-29

**Authors:** Mehdi Moustaqil, Emma Ollivier, Hsin-Ping Chiu, Sarah Van Tol, Paulina Rudolffi-Soto, Christian Stevens, Akshay Bhumkar, Dominic J. B. Hunter, Alexander N. Freiberg, David Jacques, Benhur Lee, Emma Sierecki, Yann Gambin

**Affiliations:** a EMBL Australia Node for Single Molecule Sciences, and School of Medical Sciences, Botany Road, The University of New South Wales, Sydney, Australia; b Department of Microbiology, Icahn School of Medicine at Mount Sinai, New York, NY, USA; c Department of Microbiology and Immunology, Institute for Human Infections and Immunity, The University of Texas Medical Branch, Galveston, TX, USA; d Institute for Molecular Biosciences, The University of Queensland, St Lucia, Australia; e Department of Pathology, Institute for Human Infections and Immunity, The University of Texas Medical Branch, Galveston, TX, USA

**Keywords:** SARS-CoV-2, innate immunity, protease activity, NSP3 (PLpro), NSP5 (3CLpro), IRF3, NLRP12, TAB1

## Abstract

The genome of SARS-CoV-2 encodes two viral proteases (NSP3/papain-like protease and NSP5/3C-like protease) that are responsible for cleaving viral polyproteins during replication. Here, we discovered new functions of the NSP3 and NSP5 proteases of SARS-CoV-2, demonstrating that they could directly cleave proteins involved in the host innate immune response. We identified 3 proteins that were specifically and selectively cleaved by NSP3 or NSP5: IRF-3, and NLRP12 and TAB1, respectively. Direct cleavage of IRF3 by NSP3 could explain the blunted Type-I IFN response seen during SARS-CoV-2 infections while NSP5 mediated cleavage of NLRP12 and TAB1 point to a molecular mechanism for enhanced production of cytokines and inflammatory response observed in COVID-19 patients. We demonstrate that in the mouse NLRP12 protein, one of the recognition site is not cleaved in our *in-vitro* assay. We pushed this comparative alignment of IRF-3 and NLRP12 homologs and show that the lack or presence of cognate cleavage motifs in IRF-3 and NLRP12 could contribute to the presentation of disease in cats and tigers, for example. Our findings provide an explanatory framework for indepth studies into the pathophysiology of COVID-19.

## Introduction

The ongoing pandemic of COVID-19 (Coronavirus Disease-2019) has already had a deep health, economic and societal impact worldwide [1]. COVID-19 is caused by a novel betacoronavirus, SARS-CoV-2. Other highly pathogenic betacoronaviruses include SARS-CoV and MERS-CoV, responsible for widespread outbreaks in 2002 and 2012, respectively [2].

**Figure 1.**
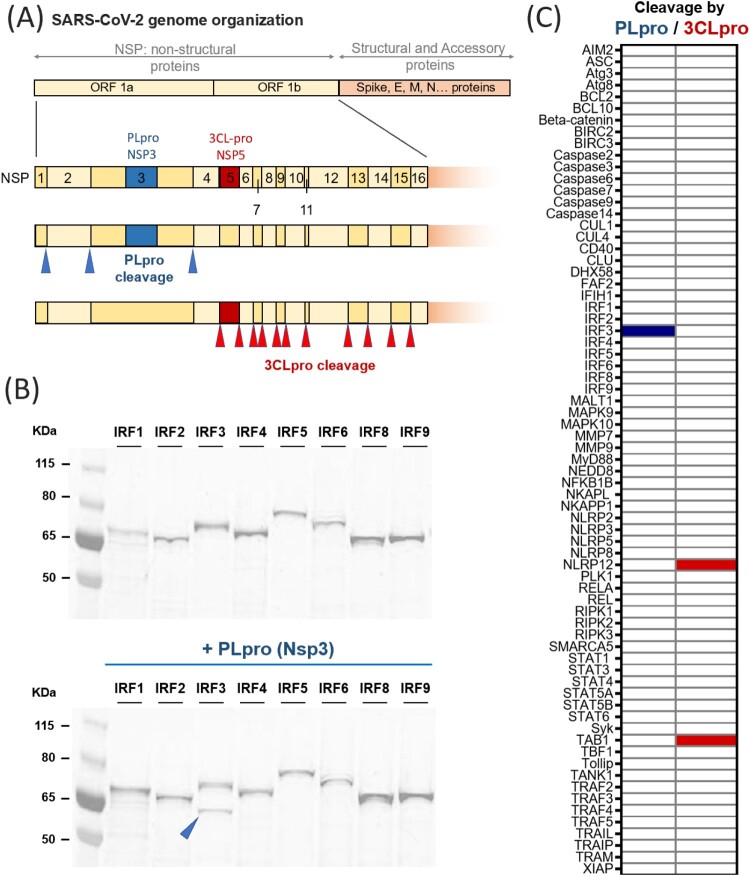
Principle of the screen of protease activity of SARS-CoV-2 PLpro and 3CLpro. (A) Schematic of the organization of the genome of SARS-CoV-2, focusing on the non-structural proteins Nsp1-16. As depicted, two proteases are encoded in ORF1a: NSP3 or papain-like protease (PLpro) and NSP5, or 3C-like protease (3CLpro). PLpro is responsible for three proteolytic cleavages, while 3CLpro cuts the large polyprotein at eleven different sites. (B) Results obtained for the family of IRF proteins ; the additional band obtained for IRF3 in the presence of PLpro indicates cleavage (C) Overview of the proteins tested in this study and the proteolytic events detected: out of the 71 proteins tested, PLpro cleaves only IRF3 (indicated in blue) and 3CLpro cleaves NLRP12 and TAB1 (as shown in red).

SARS-CoV-2 encodes a large (30 kb) single stranded, positive sense RNA genome that contains multiple open reading frames (ORFs). ORF1a and 1ab produce two large replicase polyproteins precursors (450 kDa for ORF1a, 750 kDa for ORF1ab) which upon proteolytic cleavage generates 16 non-structural proteins (NSP), 1–16. Other ORFs encode the 4 main structural proteins of SARS-CoV2: spike (S), membrane (M), envelope (E) and nucleocapsid (N) proteins, as well as accessory proteins. Processing of the polyprotein precursors relies on the two viral proteases, NSP3 and NSP5. As shown in Figure 1(A), the papain-like protease (PLpro) domain of NSP3 is responsible for the proteolytic cleavage of nsp 1-4. The protein NSP5, or 3C-like protease (3CLpro), is responsible for the processing of other cleavage sites that results in nsp 5-16. NSP4 is uniquely cleaved by NSP3 on the N-terminus and NSP5 on the C-terminus [3].

As these two proteases are essential for viral replication, they are evident drug targets. Considerable effort has been spent characterizing the structures of the protease domains of NSP3 and NSP5 [4–6], opening the door to the identification or development of inhibitors, using virtual or high-throughput screening [7–11]. Viral proteins, especially those from RNA viruses that have stricter constraints on their genome size, often perform multiple tasks. In addition to performing their intrinsic functions in the viral life cycle, many have evolved to interfere with innate immune responses or otherwise co-opt the host cell’s machinery to facilitate optimal viral replication [12,13]. Coronavirus proteases is an important determinant of viral virulence [12,14]. For example, PLpro of both SARS-CoV [15,16] and MERS-CoV [17], as well as other coronaviruses [18,19], antagonize the type I interferon (IFN) pathway via multiple mechanisms. Inactivation of different components of the pathway, including RIG-I [15], STING [15], TRAF3/TRAF6 [20], TBK1 [19] and IRF3 [16,17,21,22], has been documented. These effects are partly mediated by the protease activity but mainly derive from the deubiquitinating and deISGylating functions associated with full-length NSP3 [23–25]. SARS-CoV PLpro has also been reported to also activate TGF-β1 signalling [26] or down-regulate p53 [27]. Similarly for the NSP5 protease, 3CLpro from the feline coronavirus, feline infectious peritonitis virus (FIPV), inhibits type I interferon signalling through cleavage of NEMO [28], while the porcine deltacoronavirus (PDCoV) 3CLpro cleaves DCP1A [29]. SARS-CoV 3CLpro is responsible for virus-induced apoptosis [30]. Not surprisingly, SARS-CoV-2 PLpro and 3CLpro have already been implicated in antagonizing innate immune function [31]. Given the myriad activities ascribed to the PLpro and 3CLpro counterparts in the abovementioned orthocoronavirins, we designed a systematic screen of human innate immune pathway proteins (HIIPs), in order to better understand how SARS-CoV-2 PLpro and 3CLpro interferes with or dysregulate the host response. Our screen of 71 HIIPs revealed that only 3 proteins were directly cleaved by these two viral proteases. Notably, we discovered that PLpro directly cleaved IRF3, while 3CLpro cleaved NLRP12 and TAB1. Surprisingly, both NLRP12 and TAB1 are cleaved at two different sites, creating three protein fragments. We identified the five cognate cleavage sites in these 3 HIIPs targeted by the PLpro and 3CLpro domains of SARS-CoV-2 NSP3 and NSP5, respectively. Structure–function correlative analysis followed by comparative alignment of IRF3 and NLRP12 homologs across relevant mammalian orders reveal the potential explanatory power of our findings. The cleavage of IRF3 could explain the enigmatically blunted type-I IFN response that have been noted at early stages of SARS-CoV-2 infections, while the 3CLpro mediated cleavage of NLRP12 might explain the hyperinflammatory response observed at later stages in severe COVID-19 cases [32,33]. Indeed, the lack or presence of cognate cleavage motifs in IRF3 and NLRP12 homologs presents interesting correlations with the presentation of disease in animal models; our results will enable the development of more effective animal models for severe COVID-19. Finally, we searched the available genomes of potential hosts, to determine whether SARS-CoV-2 could have evolved into an animal where the different cleavage sites would be present. We found that out of 11 species of bats, only one presents all five cleavage sites identical to humans for NLRP12, TAB1 and IRF3. This species, *Myotis Davidii,* is found endemically in Hubei province of China, near the first epicentre of SARS-CoV-2 pandemic. We will discuss the impact of our findings for the on-going search for the reservoir host of SARS-CoV-2.

## Materials and methods

### Purification of PLpro and 3CLpro (NSP5) of SARS-CoV-2

*Escherichia coli* C41(DE3) cells were transformed with the SARS-CoV-2 pET-28a-nsp5 plasmid (IDT DNA). Single colonies were used to inoculate LB media supplemented with kanamycin (50 µg/mL). 500 mL cultures were grown at 37°C until an OD600 of 0.6 was reached, cooled to 28°C and induced for 4 h with 1 mM IPTG. Following growth, the cells were pelleted by centrifugation (5000 × g, 20 min, 4°C) and washed with 1X PBS before storage at −80°C. Frozen cell pellets were resuspended in buffer A (20 mM sodium phosphate buffer pH 8, 500 mM NaCl, 10 mM imidazole) and lysed on ice via sonication using a Branson SFX250 Sonifier (8 min at 50% amplitude, 2 s pulse on, 2 s pulse off). Cell debris was removed from the lysate by centrifugation (10,000 × g, 30 min, 4°C) and subsequent filtration of the supernatant through a 0.22 µm syringe filter. 20 mL of the clarified lysate was loaded onto a 1 mL HiTrap IMAC Sepharose FF column (GE Healthcare, Illinois) charged with Ni2+ and preequilibrated with buffer A. Unbound proteins were removed from the column through washing with 10 column volumes (CV) of buffer A. Bound proteins were eluted with a stepwise gradient of buffer B (20 mM sodium phosphate buffer pH 8, 500 mM NaCl, 500 mM imidazole) as follows: 0–5% B, 2 CV; 5% B, 5 CV hold; 5–25% B, 2 CV; 25%B, 5 CV hold; 25–100% B, 2 CV; 100% B, 5 CV hold. Fractions containing nsp5 were exchanged and concentrated into buffer C (20 mM Tris, pH 7.4, 10% glycerol), flash frozen and then stored at −80°C. All purification steps were performed on ice or at 4°C. Protein concentrations were determined using a linearized Bradford protein assay.

### Selection of HIIPs

To screen for HIIPs that might be targeted by SARS-CoV 2 PLpro or 3CLpro, we first leveraged the systems virology and systems biology tools present in relevant databases like InnateDB [34], PathBank [35] ViPR, VirHostNet2.0, and VirusMentha to downselect a core set of HIIPs that covers almost all pathways involved in human innate immune responses. We then searched protein libraries existing in our laboratory and protein clones available from collaborators to build the library of HIIPs for this screen.

### Cloning and expression of the HIIPs

The 71 Human Innate Immune Proteins (HIIPs) listed in Figure 1(C) were cloned as GFP or mCherry fusions into dedicated Gateway vectors for cell-free expression. Open Reading Frames (ORFs) were sourced from the Human ORFeome collections, versions 1.1, 5.1 and 8.1 and transferred into Gateway destination vectors that include N-terminal or C-terminal Fluorescent proteins. Most proteins screened were expressed as N-terminal enhanced GFP fusions (vector pCellFree G03); for TAB1 and NLRP12, C-terminal GFP were also used to validate the cleavage sites. The specific Gateway vectors were created by the laboratory of Pr. Alexandrov and sourced from Addgene (Addgene plasmid # 67137; http://n2t.net/addgene:67137; RRID:Addgene_67137). Mouse NLRP12 constructs were sourced from the laboratory of Dr Kate Schroeder (IMB, University of Queensland). The HIIPs were expressed *in vitro* using a cell-free expression system derived from *Leishmania tarentolae.* This eukaryotic system enables expression of full-length proteins with minimal truncations and non-specific aggregation, for proteins up to 150 kDa in size [36]. This system has been used recently to study the folding and oligomerisation of NLRP3 proteins and the polymerization of ASC [37] or the formation of higher-order assemblies of MyD88 [38,39]. The expression is simply set-up as a one-pot reaction where the plasmid encoding the protein of interest is added to the *Leishmania tarentolae extracts* (LTE); expression occurs within 3 h at 27°C and expression yields can be evaluated by the fluorescence intensity of the GFP/mCherry tags [40].

### Detecting proteolytic cleavage of the HIIPS

The 71 HIIPs proteins were expressed individually in 10 µL reactions (1 µL DNA plasmid at concentrations ranging from 400 ng/µL to 2000ng/µL added to 9 µL of LTE reagent). The mixture was incubated for 30 min at 27°C to allow the efficient conversion of DNA into RNA. The samples were then split into controls and protease-containing reactions. The proteases PLpro (nsp3) and 3CLpro (nsp5) were added at various concentrations, and the reactions were allowed to proceed for another 2.5 h at 27°C before analysis.

The controls and protease-treated LTE reactions were then mixed with LDS (Bolt LDS Sample Buffer, ThermoFisher) and loaded onto SDS-page gels (4–12% Bis-Tris Plus gels, ThermoFisher); the proteins were detected by scanning the gel for green (GFP) or red (mCherry) fluorescence using a ChemiDoc MP system (BioRad) and proteolytic cleavage was assessed from the changes in banding patterns, as shown in Figure 1(B). Note that in this protocol, the proteins are not treated at high temperature with the LDS and not fully denatured, to avoid destruction of the GFP/mCherry fluorescence. As proteins would retain some folding, the apparent migration on the SDS-page gels may differ slightly from the expected migration calculated from their molecular weight. We have calibrated our SDS-page gels and ladders using a range of proteins, as shown in Supplementary Information.

### Preparation of LTE system

*Leishmania tarentolae* extracts were prepared in house using the protocol described previously [36]. Briefly, *Leishmania tarentolae* Parrot strain was obtained as LEXSY host P10 from Jena Bioscience GmbH, Jena, Germany and cultured in TBGG medium containing 0.2% v/v Penicillin/Streptomycin (Life Technologies) and 0.05% w/v Hemin (MP Biomedical). Cells were harvested by centrifugation at 2500 × g, washed twice by resuspension in 45 mM HEPES, pH 7.6, containing 250 mM Sucrose, 100 mM Potassium Acetate and 3 mM Magnesium Acetate and resuspended to 0.25 g cells/g suspension. Cells were placed in a cell disruption vessel (Parr Instruments, USA) and incubated under 7000 KPa nitrogen for 45 min, then lysed by rapid release of pressure. The lysate was clarified by sequential centrifugation at 10 000 × g and 30 000 × g and anti-splice leader DNA leader oligonucleotide was added to 10 μM. The lysate was then desalted into 45 mM HEPES, pH 7.6, containing, 100 mM Potassium Acetate and 3 mM Magnesium Acetate, supplemented with a coupled translation/transcription feeding solution and snap-frozen until required. We verified that the expression patterns and cleavage of the proteins in this study was independent of the batch of LTE used.

### Western blot and antibodies used

For Figure 5 panels A,B and C: 293T-ACE2 cells were lysed with RIPA lysis buffer (Pierce) containing a cocktail of protease inhibitors (Cell Signaling). Equivalent amounts of proteins determined by the Bradford protein assay (Bio-Rad) were separated by SDS-PAGE and transferred through the Trans-Blot Turbo transfer system (Bio-Rad). To avoid the nonspecific antibody reaction, the membranes were blocked with Intercept Blocking Buffer (LI-COR) prior to the addition of primary antibodies. After primary antibodies incubation, the blots were then treated with Alexa Fluor 647 conjugated secondary antibodies (Invitrogen) and developed signals using the ChemiDoc MP image system (Bio-Rad). The following commercial antibodies were used: anti-IRF3 (ab68481) and anti-ACE2 (ab108252) from Abcam, anti-NLRP12 (PA5-21027) from Invitrogen, anti-TAB1 (#3226) from Cell Signaling, anti-SARS-CoV-2 N (GTX632269) from Genetex, anti-COXIV (11242-1-AP) from Proteintech.

For Figure 5 panels D–F and G–I: 293T-ACE2 (800,000) cells were plated on 6-well plates in 2 mL of media (10% FBS DMEM (Corning)) overnight at 37°C and 5% CO_2_. Eighteen hours after plating, the cells were mock-treated, transfected (Lipofectamine 2000, Invitrogen) with low molecular weight (LMW) poly(I:C) (1 μg/mL) (InvivoGen), or infected with an infectious clone of SARS-CoV-2 expressing GFP (icSARS-CoV-2 mNeonGreen) [41] at multiplicity of infection (MOI) 0.3 or 3.0 for one hour at 37°C and 5% CO_2_ with rocking every 15 min. At the end of the hour incubation, cells were washed once with 1 mL of 1× PBS to remove the virus inoculum. After washing, fresh 2% FBS DMEM was added and the cells were incubated at 37°C and 5% CO_2_ until sample collection at 6, 24, or 48 h post-infection. At each time point, bright field and GFP fluorescence images were taken (Olympus IX83 inverted microscope) and protein lysates were collected. Protein lysates were collected in 2X Laemmli with 5% beta-mercaptoethanol (β-ME) and boiled at 95°C for 20 min prior to removal from the BSL-4. All work with infectious virus was carried out under biosafety level 4 conditions in the Galveston National Laboratory at the University of Texas Medical Branch (UTMB). For immunoblotting, protein lysates were run on 4–15% SDS-PAGE gels and transferred onto a methanol-activated polyvinylidene difluoride (PVDV) membrane (Bio-Rad). The following antibodies were used: NALP12 (NLRP12) (1:1000) (Invitrogen, PA521027), IRF3 (1:1,000) (Cell Signaling Technology (CST), 4302S), TAB1 (1:1000) (CST, 3226S), SARS-CoV/SARS-CoV-2 nucleocapsid (1:1000) (GeneTex, GTX632269), and tubulin (1:2000). Immunoblots were developed with the following secondary antibodies: enhanced chemiluminescence (ECL) anti-rabbit IgG horseradish peroxidase (HRP)-conjugated whole antibody from donkey (1:10,000) (NLRP12, TAB1, and IRF3) and ECL anti-mouse IgG HRP-conjugated whole antibody from sheep (1:10,000) (SARS-CoV nucleocapsid, tubulin) (GE Healthcare). The proteins were visualized with either Pierce or SuperSignal West Femto luminol chemiluminescence substrates (Thermo Scientific). For densitometry analysis, the amount of protein expressed was determined using ImageJ to calculate the area under the curve (AUC). Protein expression was normalized as follows: ((IRF3 or NLRP12 or TAB1 AUC sample/IRF3 or NLRP12 or TAB1 AUC mock)/(tubulin sample/tubulin mock)), as shown in Figure S14.

Alignment of cleavage sites (Figure 6 and Figure 8):

Protein sequences for Cotton rats (Sigmodon hispidus) and Minks (Neovison vison) were found using tblastn against shotgun genomes, with the Query AAH7172.1 for IRF3 protein [(Isoform 1) Homo sapiens] and NP_653288.1 for NACHT, LRR and PYD domains-containing protein 12 [(isoform 2) Homo sapiens]. Cleavage sites were mapped using NCBI alignment tools and compiled for the figures.

## Results

### An *in-vitro* protease assay identifies targets of SARS-CoV2 PLpro and 3CLpro

Figure 1(A) shows the location of NSP3 (PLpro) and NSP5 (3CLpro) in the SARS-CoV-2 genome as well as the cognate proteolytic sites targeted by PLpro and 3CLpro. These proteolytic sites serve as references for the motif analysis in cognate HIIPs identified in our screen that are cleaved by either PLpro or 3CLpro.

We gathered the Open Reading Frames of 71 different HIIPs, selected to include the major proteins associated with the signalling pathways of innate immunity and cell death such as proteins downstream of the nucleic acid sensors MDA-5 and RIG-I (e.g. TRAF3, NFκB and IRFs), effectors of the Toll-like receptors, TLR3 and TLR7, such as TRIF, TRAM, TRAF6 or TAB1, and effectors of cell-death (e.g. TRAF2, caspases, Bcl2, XIAP).

These 71 human proteins were cloned for expression as GFP-fusions in a cell-free expression system based on the eukaryotic organism of *Leishmania tarentolae* (LTE)*.* This system produces full-length proteins up to 200 kDa with minimal truncations, minimal protein aggregation and was previously used by our group to study the behaviour of various apoptotic proteins such as MyD88 [38], MAL [39] or ASC and NLRP3 [42].

The assay was designed as a one-pot reaction to rapidly identify proteolytic cleavage. Purified recombinant protease domains were added to the LTE during expression of the target proteins (see Supplementary Figure 1). The screening conditions were optimized to avoid off-target effects and false positives. The human proteins targets were typically expressed at low concentration (reaching at most 1 μM), in a crowded environment (LTE) that recapitulates the host cytosol. The proteases were allowed to react to the *de novo* synthesized target protein for about 2½ h, at 27°C (optimal temperature for protein expression using LTE). Under these conditions, it is probable that the activity of the proteases was greatly reduced.

We used the GFP-tag on the target protein to directly visualize cleavage using reducing SDS-PAGE (Figure 1(B)). As expected, partial denaturation (i.e. no thermal denaturation) maintained the fluorescence of GFP so that proteins could be imaged without any subsequent purification steps. Comparing the protein migration patterns in the presence and absence of the protease identifies cleavable proteins. Indeed, an intact protein would appear on the gel as a single fluorescent band. If the protein is cleaved, then the gel will show either a single band, at a lower molecular weight (in the event of a complete proteolysis of all target proteins), or multiple fluorescent bands, corresponding to the full-length protein and its cleavage product in the case of an incomplete cleavage process, as described in Figure 1(B). The use of a fluorescent tag also allows simple quantification of protein concentration based on fluorescence intensity.

SDS-PAGE showed no difference of sizes in the presence or absence of viral proteases for most HIIPs tested, indicating that a large majority of HIIPs were unaffected by the addition of PLpro and 3CLpro. This suggests that in our assay conditions, non-specific cleavage was not observed. However, 3 cases of proteolytic degradation were identified, giving confidence that the viral proteases are active (Figure 1(C)). The fact that only specific members of the same family of proteins (e.g. IRFs, Figure 1(C)) were cleaved (IRF3 cleavage by PLpro) suggests high specificity and the recognition of specific sequences. There was no common reactivity between PLpro and 3CLpro reinforcing the idea that each viral protease did indeed recognize a specific consensus sequence (Figure 1(C)).

The screen results also revealed that protein expression levels were unchanged upon addition of the proteases, suggesting that none of the components required for cell-free expression were cleaved during the experiments. As shown in Supplementary Figure 2, the cell-free lysate acts as a crowded environment made up of many different proteins. Analysis of the Coomassie stained gels shows that staining intensity and profile were similar, even at the highest PLpro concentration, indicating that there was no significant cleavage of components of the cell-free reagent.

### PLpro selectively cleaves IRF3

**Figure 2.**
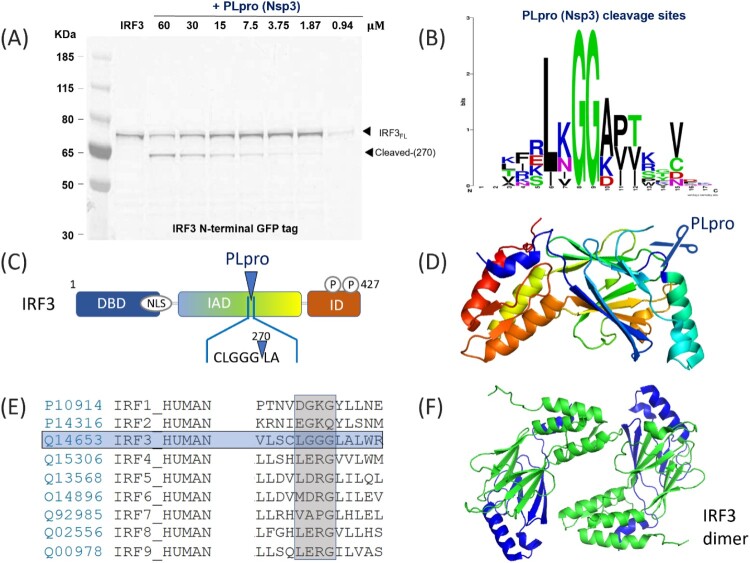
Cleavage of IRF3 by SARS-CoV-2 PLpro. (A): SDS-page analysis of the cleavage of human IRF3 protein, with a N-terminal GFP tag. The protein was expressed alone or in the presence of increasing concentrations of the SARS-CoV-2 protease PLpro. (B) Logo analysis of the cleavage site predicted from the polyprotein cleavage of SARS-CoV-2. (C) Representation of the domains found in IRF3 and the position of the cleavage site. (D) Representation of IRF3 structure (from PDB 1J2F). The cleavage sequence LGGG is highlighted in blue (E) Alignment of the amino acids for human IRF1-IRF9, demonstrating that only IRF3 would be predicted to be cleaved as observed. (F) Structure of the IRF3 homodimer (from PDB 1QWT), showing the two fragments (green, N terminal, green, and blue for C-terminal).

To further validate that PLpro could cleave IRF3, we titrated different concentrations of the protease in the reaction. As shown in Figure 2(A), a strong concentration-dependence was observed, as expected. When the same experiment was performed in the presence of 3CLpro, no proteolysis was detected, validating that the cleavage is indeed specific to PLpro (Supplementary Figure 3).

We then set out to identify the cleavage site on IRF3. Based on the proteolysis sites on ORF1a and ORF1ab [3], and similarly to SARS-CoV PLpro and MERS-CoV PLpro, SARS-CoV-2 PLpro recognizes and cleaves after LXGG sequences (Figure 2(B)). We found a single LGGG sequence at residues 268–271 of the canonical isoform of human IRF3. Cleavage of the N-GFP tagged protein at this site would result in the formation of a GFP-tagged 57 kDa fragment (30 kDa + 27 kDa for the GFP) and a 17 kDa untagged fragment, which corresponds well to the band obtained by SDS-PAGE (Figure 2(C)). The identified cleavage site would be present on an exposed loop, based on previously solved structures (see Figure 2(D), PDB: 1J2F) and therefore accessible to the protease. No such motif was found in any other member of the IRF family (Figure 2E), in agreement with our data. On the contrary, recognition motifs present in other proteins of our test panel (LAGG in β-catenin, LVGG in STAT5A and LEGG in NLRP12) did not get processed by PLpro in our assay. In the case of STAT5A, the cleavage motif is partially buried in the protein (see Supplementary Figure 4). However, the LAGG motif in β-catenin is exposed at the surface in a structured a-helix (see Supplementary Figure 5); the LEGG motif in NLRP12 cannot be located on the only existing structure NLRP (NLRP3, PDB 6NPY). It is possible in addition to local structures, the residue between L and GG would contribute to the selectivity of PLpro cleavage.

IRF3 is a key mediator of type I IFN response triggered by viral infections [43]. The C-terminal part of the protein is responsible for mediating interactions with upstream receptors and effectors STING, MAVS and TRIF [44]. IRF3 tail is also strongly targeted for post-translational modifications upon infection [45,46], leading to its homodimerization (PDB: 1QWT, Figure 2(F)), translocation into the nucleus and transcriptional activation [47]. Therefore, we reasoned that PLpro cleavage of IRF3 would result in reduced IFN production, a feature that has been observed upon SARS-CoV-2 infection [48].

### 3CLpro Cleaves TAB1 and NLRP12

Similarly, we set out to validate 3CLpro proteolysis of TAB1 and NLRP12 (Figure 1(C)). As before, we observed a concentration-dependent cleavage of both proteins and verified that PLpro did not have an effect, at any concentration (see Supplementary Figure 6 and 7).

**Figure 3.**
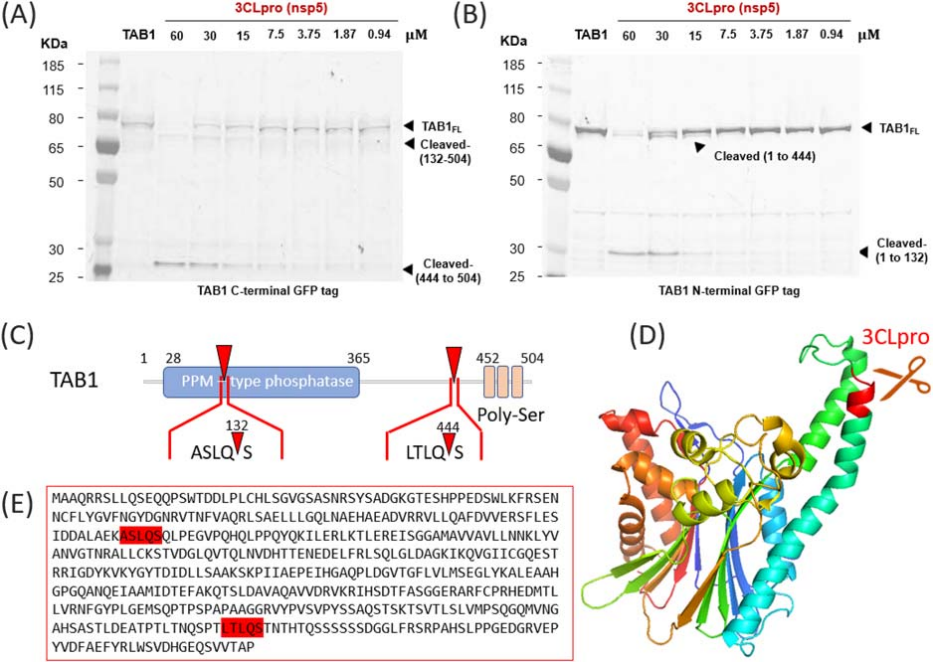
3CLpro (Nsp5) cleaves TAB1 at two separate sites. A): SDS-page analysis of the cleavage of human TAB1 protein, with a C-terminal GFP tag. The protein was expressed alone or in the presence of increasing concentrations of the SARS-CoV-2 protease 3CLpro. The gel shows two additional bands upon cleavage, corresponding to the fragment 132-504 and to the fragment 444 to 504.. (B) same, but for the N-terminal GFP. In this case, the fragments 1-132 and 1-144 are fluorescent and can be detected on the gel, while the fragments 132-444, 444-504 and 132-504 are not fluorescent. (C) Schematic representation of TAB1 protein structure with the location of the identified cleavage sites on the primary sequence. (D) Representation of TAB1 structure (from PDB 2POM). The cleavage sequence ASLQS is highlighted in red. (E) full sequence of amino acids for human TAB1, showing the two putative cleavage sites ASLQS and LTLQS.

SDS-PAGE analysis reveals the presence of two cleavage sites on TAB1, that can be more easily visualized when the GFP tag is placed at either the C-terminus (Figure 3(A)) or the N-terminus (Figure 3(B)) of the protein. The recognition motif for 3CL-Pro of coronaviruses SARS-CoV, MERS-CoVand SARS-CoV-2 [49] is often LQ/(S,A,G). In TAB1, we identified a LTLQS motif at position 441 of the canonical form, that would give rise to a 48 kDa N-terminal fragment and a 6 kDa C-terminal fragment. Another possible recognition motif (ASLQS) is present at position 129 and that would give a 14 kDa N-terminal fragment and a 40 kDa C-terminal fragment (Figure 3(C)). Therefore, the two proteolytic fragments for C-GFP TAB1 correspond to amino-acids 133–504 and 445–504 (Figure 3(A)) whereas N-GFP TAB1cleavage leads to the formation of proteolytic fragments corresponding to residues 1–132 and 1–444 (Figure 3(B)). More details on calculation of sizes based on migration are included in Supplementary Figure 8 and 9. Based on the reported structure of TAB1 (Figure 3(D), PDB: 2J4O), the first cleavage site is on an exposed loop in the pseudo-phosphatase domain. The second cleavage site is in a disordered region of the protein that does not appear on the structure (see Figure 3(E) for the full sequence of human TAB1.)

Another target of 3CLpro revealed by our screen is NLRP12. NLRP12 is an intracellular pattern-recognition receptor, from the nucleotide-binding and oligomerization domain-like (NLR) receptor family, which regroups key mediators of the innate immune response and inflammation [50]. NLRP12 modulates the expression of inflammatory cytokines through the regulation of the NFκB and MAPK pathways [51]. Formation of NLRP12 inflammasome can activate caspase 1 [52], which produces interleukin 1β and 18 and leads to cell death. But NLRP12 can also form mixed inflammasomes with other NLRPs, including NLRP3, and then play an inhibitory role [53,54]. NLRP12 is also involved in adaptative immunity and controls MHC class I expression through a yet ill-defined mechanism [55].

**Figure 4.**
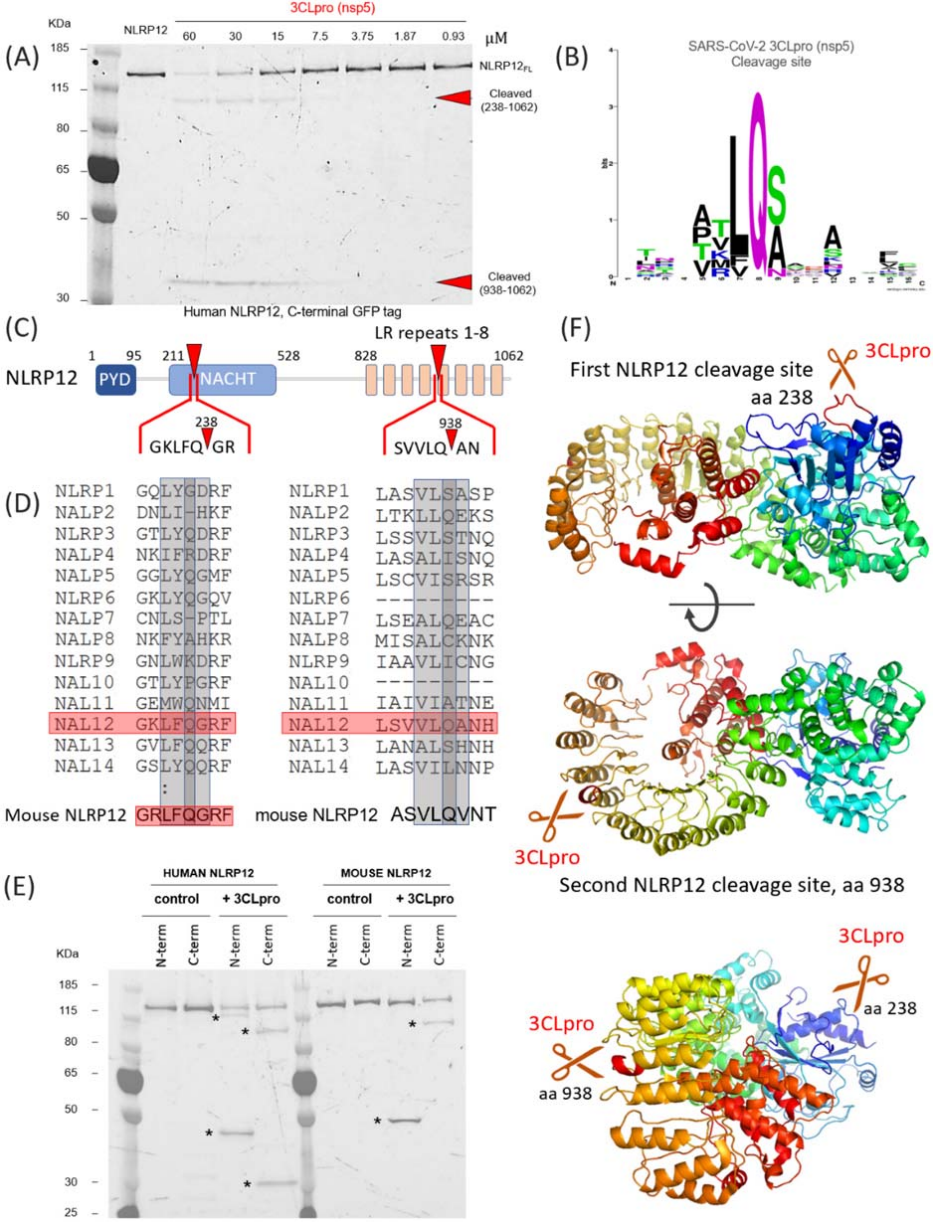
Cleavage of NLRP12 by 3CLpro of SARS-CoV-2. (A): SDS-page analysis of the cleavage of NLRP12 protein, with a C-terminal GFP tag. The protein was expressed alone or in the presence of increasing concentrations of the SARS-CoV-2 protease 3CLpro. (B) Logo analysis of the cleavage site predicted for 3CLpro, from the polyprotein cleavage of SARS-CoV-2. (C) Representation of the domains found in NLRP12 and the position of the cleavage sites.(D) Alignment of the amino acids for human NALPs (NLRPs), demonstrating that only NLRP12 would be predicted to be cleaved as observed. Below, alignment of mouse NLRP12, showing that the first cleavage site is conserved, but the second site presents an A→V mutation that would disrupt cleavage. (E) SDS-page analysis of human and mouse NLRP12, with different tag orientations, to demonstrate the differences between species. The banding patterns obtained in the presence of 3CLpro are consistent with the predicted sizes. Using the mouse NLRP12 constructs, only one cleaved fragment is observed in the N-term and C-term constructs. (F) Representation of NLRP12 structure (derived from the structure of NLRP3 from PDB 6NPY). (top): The cleavage sequence LFQG (site 1, at residue 238) is highlighted in red; the site is presented in a flexible loop and seems fully exposed for cleavage by 3CLpro. (middle): The second cleavage site, (LQA) found at residue 938, it presented in a flexible loop and would be accessible at the tip of an alpha helix in the LR repeats. (bottom): in this view, both cleavage sites are visible.

Unexpectedly, we also noted the presence of two additional bands when NLRP12 was subjected to 3CLpro treatment, suggesting that two cleavage sites would also be found in NLRP12 (Figure 4(A)). In Figure 4(B), we show the analysis of preferred residues for cleavage by 3CLpro of SARS-CoV-2. We identified a canonical LQA motif at residue 938, at the C-terminal of NLRP12, in the middle of the LR repeats (Figure 4(C)). The cleavage at residue 938 would create a small C-terminal fragment (residues 938-1062), observed on the gel using the C-terminally GFP-tagged NLRP12 (Figure 4(A)). There was no other canonical 3CLpro recognition sequence (LQS) in NLRP12, to explain the second proteolytic event. However, it was shown that 3CLpro can also cut after FQ or VQ motifs, and that presence of a combination of hydrophobic and positively charged residues at position P-3 and P-4 seems to be preferred (Figure 4(B)). When manually scanning the NLRP12 sequence for degenerated pairs (FQ/VQ), we identified a KLFQG sequence at residue 238 (Figure 4(C)). Cleavage at this site would result in the formation of a 93 kDa C-terminal fragment (residues 241-1,062), observed in Figure 4(A). Analysis of the sequences of other NLRPs (NLRP1-NLRP14) shows that both motives are unique to NLRP12 (Figure 4(D)). Interestingly, the mouse homolog of NLRP12 possesses the first but not the second recognition motif (Figure 4(D)). To further validate our data, we compared the effect of 3CLpro on human vs. mouse NLRP12, each tagged at the N- and C-terminal position. Figure 4(E) shows that human NRLP12 (left panel) is indeed cleaved twice, whereas mouse NLRP12 (right panel) is only processed once. Here, a myc-mCherry was used and the tag was detected by the red fluorescence of mCherry. In the N-term configuration, a single cleaved product is detected, corresponding to the fragment 1–238. In the C-term configuration, the fragment 238–1062 is detected; this confirms that the LQA→LQV mutation found in mice inhibits cleavage by 3CLpro. The calibration of migration of the fragments on the SDS-page gels is further described in Supplementary Figure 10. Homology modelling of NLRP12, using the structure of its close relative NLRP3 (PDB: 6NPY), shows that both sites are expected to be in an exposed, unstructured loops, readily accessible to a protease (Figure 4(F)).

### Decrease of IRF3, TAB1 and NLRP12 levels in SARS-CoV-2 infected cells

To validate the cleavage of IRF3, TAB1 and NLRP12 in SARS-CoV-2 infected cells, we conducted two infection experiments in two separate laboratories to study the expression levels of these proteins. Similar to SARS-CoV, SARS-CoV-2 utilizes ACE2 to achieve its entry step. Therefore, we generated ACE2 expressing 293T cells by lentiviral expression system for enhancing the infectivity of SARS-CoV-2.

**Figure 5.**
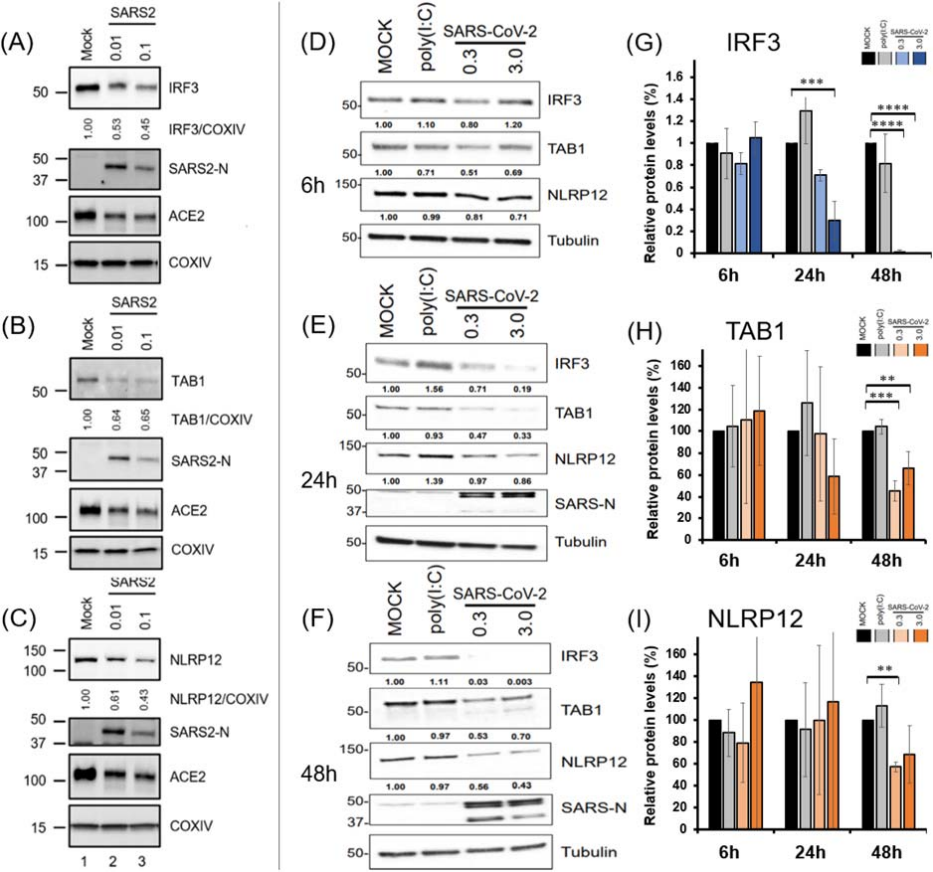
IRF3, TAB1 and NLRP12 are decreased upon infection with SARS-CoV-2 in 293T-ACE2 cells, in two independent laboratories. (A–C) 293T-ACE2 cells were infected with SARS-CoV-2 and analysed for viral and host protein levels 72 h post-infection. (D–I) Independently, stable 293T-ACE2 were infected with icSARS-CoV-2 mNeonGreen) and the levels of IRF3, TAB1 And NLRP12 were visualized by Western Blotting at 6 h (D), 24 h (E) and 48 h (F).(G–I): Protein levels measured by densitometry for IRF3 (G), TAB1 (H) and NLRP12 (I) and plotted for the Mock, poly(I:C) and SARS-CoV-2 infection at 6 h, 24 h and 48 h.

In the first laboratory (Mount Sinai, NYC, USA), Stable 293T-ACE2 cells generated by lentiviral transduction were infected with SARS-CoV-2 at 0.01 or 0.1 MOI. Uninfected cells plated and treated identically served as mock-infected controls. 72 hpi, cell lysates were collected, and the indicated proteins were detected by western blot using the relevant antibodies as specified in methods. The relative amounts of IRF3, TAB1 and NLRP12 were quantified by densitometry and presented as a ratio of COXIV after normalization with mock infection conditions. Figure 5 shows that the expression of IRF3, NLRP12, and TAB1 (panels A–C, respectively) was decreased in virus-infected cells (lanes 2–3) compared to the mock-infected controls (lanes 1).

In a separate set of experiments, in the Biosafety level 4 facility of the Galveston National Laboratory at the University of Texas Medical Branch (UTMB), we performed kinetic experiments. We infected 293T-ACE2 cells with icSARS-CoV-2 mNeonGreen, at two different MOI (0.3 or 3.0). Samples were collected at 6, 24 and 48 h post-infection, and compared with mock infection or transfection of 1ug/mL LMW poly(I:C). The mNeonGreen system enables visualization of the infection levels in the 293T-ACE2 cells, as shown in Figure S12 and S13. Endogenous protein levels of IRF3, TAB1 and NLRP12 were analysed by Western Blotting, as shown in Figure 5 (panels D–F). The results show a dramatic reduction of IRF3 starting at 24 h post-infection, with non-detectable levels at low MOI at 48 h post-infection. TAB1 was also significantly reduced in high and low MOI at 48 h, and NLRP12 is significantly reduced in the low MOI at 48 h. Experiments were repeated three times independently with similar results, and data in G, H, I are presented as relative protein levels (percent of Mock) ± S.D (*n* = 3). Western Blots and relevant cell imaging can be found in Supplementary materials (Figure S11–S14).

Overall, we found a robust and reproducible reduction of the protein levels of IRF3, TAB1 and NLRP12 in different cell lines, and in two separate laboratories. As our experiments probe the total amount of IRF3, TAB1 and NLRP12 in all cells, a significant reduction of protein levels would only be detected if a large number of cells are infected. Our results at MOIs of 0.01, 0.1, 0.3 and 3 show that the overall reduction of protein levels correlates with infection levels, as expected.

Combined with the results from *in vitro* assay, we found that the proteases of SARS-CoV-2 (PLpro and 3CLpro) can degrade IRF3, TAB1, and NLRP12, potentially leading to the imbalance responses of host innate immunity.

**Figure 6.**
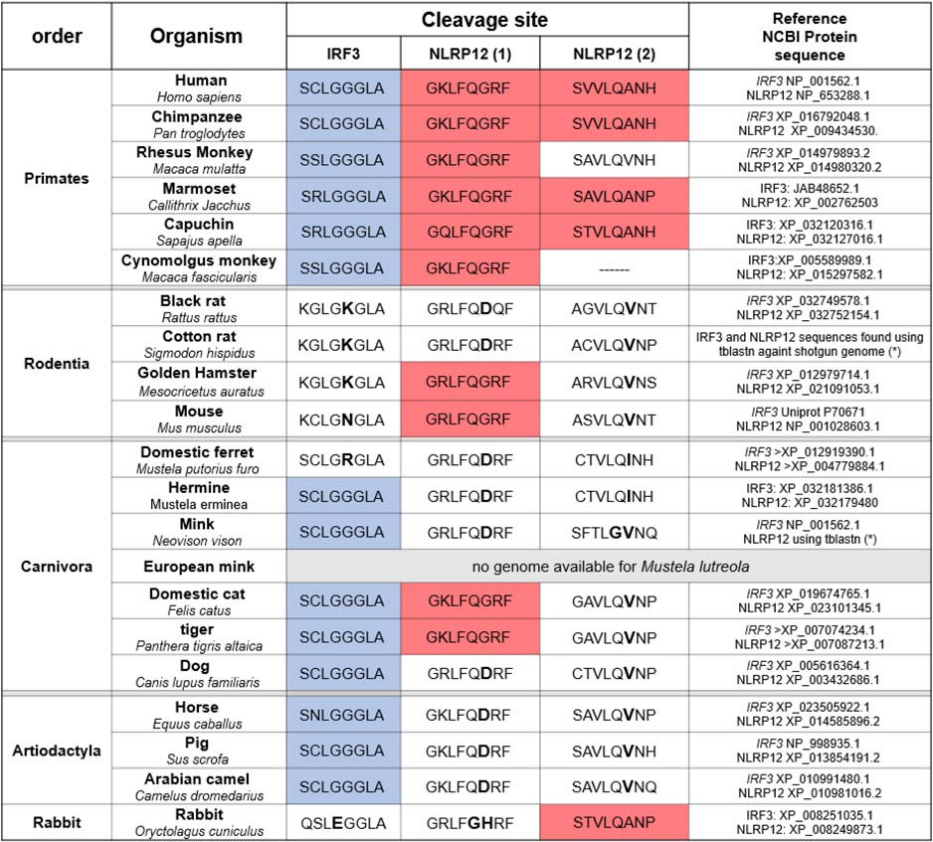
Analysis of the protein sequences across species for IRF3 and NLRP12 cleavage sites. (*) see Materials and Methods for protein sequences for Cotton rats (*Sigmodon hispidus*) and Minks (*Neovison vison*).

To validate that the observed decreases in protein levels are due to proteolytic cleavage, we used mutants of PLpro and 3CLpro that have been shown to have inhibit protease activity, as shown in Figure 6. For PLpro, we used the two point-mutants C112A, and D287A, and compared the impact on IRF3 protein levels. 293T cells were co-transfected with mCherry-IRF3 and WT, C112A or D287A PLpro, or empty vector, and levels of IRF3 and PLpro constructs were determined by Western Blotting, as described above. The data show that transfection of catalytically dead mutants of PLpro does not result in significant decrease of IRF3 levels, demonstrating that the decrease of IRF3 observed in linked to the protease activity of PLpro.

To demonstrate that 3CLpro cleaves actively TAB1, we used again two point-mutants of 3CLpro, H41A and C145A, with reduced proteolytic activity. WT and mutant 3CLpro domains were co-transfected with TAB1 in 293T cells, and protein levels were measured by Western Blotting. When WT 3CLpro is used, the TAB1 band is no longer visible, but the 3CLpro mutants do not cause a significant reduction of the levels of TAB1, despite expressing at much higher levels than WT 3CLpro. These results demonstrate that the decrease of TAB1 levels is due to the proteolytic activity of 3CLpro of SARS-Cov-2.

### Analysis of PLpro and 3CLpro cleavage sites across species

The recognition sequences on IRF3 and NLRP12 are unique in their respective families of proteins, so we wanted to investigate how conserved these motifs were. To this end, we compared the protein sequences of IRF3 and NLRP12 among species, specifically around these sequences. In both cases, the cleavage sites were located on well conserved portions of the proteins but small differences at or near the cleavage site were identified. Figure 6 summarizes the conservation of the IRF3 and NLRP12 sequences for 21 species that could be use as infection models. All primates tested presented both IRF3 and NLRP12 cleavage sequences, except for the Rhesus Monkey and Cynomolgus (“crab-eating”) monkey where the second NLRP12 cleavage site is mutated or missing. The PLpro recognition sequence in IRF3 is present in most species, except for rodents and ferrets, where the G at P2 is replaced by K, R or N, which would not be permissive to proteolytic cleavage.

We then examined the sequences of NLRP12. The motif around the first cleavage motif is mainly conserved, but we noticed that the amino acid directly after the cleavage site varied significantly. In primates, this amino acid is a small neutral amino acid (G) that is replaced by a bulkier, charged residue D or H in other species. A similar trend is observed for the second cleavage site where the small amino acid A found in human is replaced by larger amino acids (V or I). Such substitutions would likely affect the electrostatic environment and most likely inhibit the formation of active enzyme–substrate complexes. Interestingly, apart from primates, only cats and tigers have 2 similar recognition sequences to humans, for both proteins. Ferrets have variations in the cleavage sites, and it is unlikely that the proteins would be affected. Horses, pigs and camels possess the IRF3 cleavage site but not the NLRP12 sites; on contrary, the rabbit has a mutation in IRF3, but we would predict that the second NLRP12 site would be cleaved. Both cleavage sites of TAB1 are exactly conserved across all these species.

It has been reported that, besides primates, cats are amongst the few species that could not only be infected with SARS-CoV-2, but develop COVID-19 clinical signs. Anecdotal evidence suggest that Amur tigers (*Panthera tigris altaica*) and European minks *(Mustela lutreola)* could infect/be infected by humans and develop signs of illness. Unfortunately, the genome annotation for European minks is incomplete and does not allow for a comparison of its NLRP12 sequence.

## Discussion

### Experimental screens identify accessible cleavage sites in proteins and more importantly, detects non-canonical sequences

In this report, we show that the viral proteases PLpro and 3CLpro of SARS-CoV-2 lead to the *in-vitro* proteolytic cleavage of three important proteins of the host immune response: IRF3, TAB1 and NLRP12 (Figure 1(B)). These results first show the exquisite specificity of the viral proteases, with only 3 positive hits observed out of 142 experiments.

In the case of PLpro, the recognition sequence seems well defined and LXGG motifs are scarcely found in the proteins we studied. Nevertheless, of the 4 proteins that harbour such motifs, only 1 (IRF3) was cleaved *in vitro*. Indeed, the motif is present on an unstructured loop of IRF3 that made this site readily accessible. This shows that the presence of a recognition motif is required but not sufficient to predict biological activity. As such, experimental validation of potential targets identified by bioinformatics remain critical.

The identification of NLRP12 and TAB1 as substrates of 3CLpro further demonstrate the importance of this type of screening approaches. 3CLpro recognition motif is not as well defined as PLpro, and LQ/(S,A,G) motifs are ubiquitously found in the proteome. In our protein list, multiple *bona fide* recognition motifs based on LQ/S, LQ/A and LQ/G, respectively, can be found as well as several degenerated motifs. Yet only two targets (and four cleavage sites) were recognized by 3CLpro, emphasizing again the importance of site accessibility. More importantly, our data identify an unexpected cleavage site on NLRP12 (KLFQ/G) that does not resemble to the cleavage motifs present on the virus ORF1a/1ab, further indicating that the determinants of selectivity for 3CLpro are yet to be identified.

### IRF3, TAB1 and NLRP12 are important components that drive the inflammatory response to SARS-CoV-2 infection

**Figure 7.**
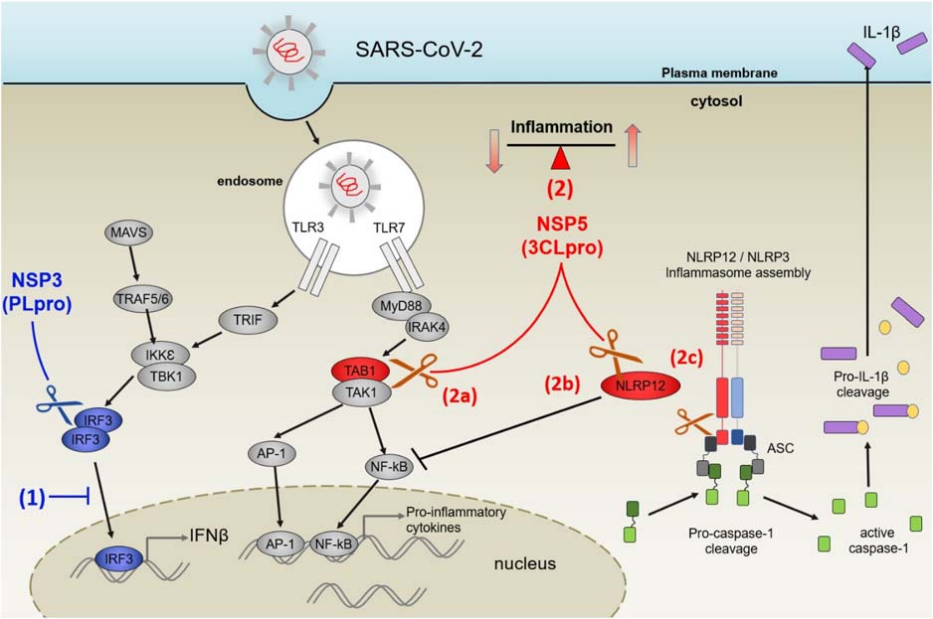
PLpro and 3CLpro of SARS-CoV-2 interfere with innate immune response by directly cleaving IRF3, TAB1 and NLRP12. (1) In blue: PLpro (Nsp3) inhibits IFNβ production by cleaving IRF3. (2) in red: 3CLpro could interfere with production of pro-inflammatory cytokines at two levels: cleavage of TAB1 would inhibit activation of NF-κB via TAK1 (2a), while cleavage of NLRP12 could release its inhibitory effect on NF-κB (2b). In addition, the cleavage of NLRP12 by 3CLpro could perturb the NLRP3 inflammasome assembly (2c), especially as one of the cleavage sites would release a PYD domain from NLRP12. This could trigger the cleavage of pro-Caspase-1 and enhance the release of IL-1β.

Viral proteases have probably evolved to efficiently process their own polypeptides but their ability to target host proteins, especially the ones involved in host defence, provide them with an evolutionary advantage. The three HIIPs identified in this screen (IRF3, TAB1 and NLRP12) are important contributors to the innate immune and inflammatory responses, either driving or dampening it, as shown in Figure 7.

IRF3 belongs to the interferon-regulatory factor (IRF) family. All IRFs possess a well-conserved DNA-binding domain at the N-terminus and a variable C-terminal domain that mediates most of the interactions with the other IRF proteins and other co-factors [56]. Fine-tuning of the choice of partners rely on post-translation modifications, such as phosphorylation and ubiquitination, in this region. As mentioned before, IRFs, especially IRF1, 3, 5 and 7, are major contributors to the production of and response to type I IFNs, which stimulate macrophages and NK cells to elicit anti-viral responses [47]. Therefore, IRF3 has been found to be targeted by several different classes of viruses. Paramyxoviruses, herpesviruses, reovirus and double stranded RNA viruses, to cite some examples, have been shown to interfere with IRF3 signalling through different strategies [57]. These include the control of protein expression, protein cellular localization, modifications of the PTMs, inhibition of protein–protein interactions or induced cellular degradation [13]. Direct proteolytic cleavage of IRFs by viral proteases has also been identified before. Proteases of enteroviruses EV-A71 [58] and EV-D68 [59] have been shown to directly cleave IRF7.

Papain-like protease domains of several coronaviruses including SARS-CoV and MERS-CoV have been implicated in the observed reduction of IFN signalling upon viral infection [60]. Although this link seems well established, the molecular basis of this effect is still under debate. Besides its proteolytic activity on the Orf1a/b, PLpro of *Coronaviridae* also possess a deubiquitinase and deISGylation activity that contribute to inhibition of IRF3 activity [16,21,22,44,61]. Recently, it has been shown that PLpro of SARS-CoV-2 cleaves the ubiquitin-like protein ISG15 [31]. Interestingly, while PLpro of SARS-CoV-2 and SARS-CoV have 83% sequence identity, the same study demonstrated significant differences in substrate specificity: PLpro of SARS-CoV exhibits preference for K48-linked di-ubiquitin, while PLpro of SARS-CoV-2 has higher reactivity towards ISG15. It was then shown that PLpro of SARS-CoV-2 causes a reduction in ISGylation of host proteins, including IRF3, to dysregulate anti-viral immune response [31].

If the activity of PLpro on the L*GG motif found in Ubiquitin, ISG and NEDD8 was previously known, we discovered that the same L*GG motif is present in the IRF3 sequence, in a loop exposed at the surface of the IRF3 protein. Here we show that *in-vitro*, SARS-CoV-2 PLpro was able to directly cleave IRF3 and validated that the effect could be observed in relevant virus infected cells (Figure 5). That NSP3 would act on the IRF3 pathway at two levels (indirect via ISG15 and direct cleavage) likely ensures that the type I interferon production is completely suppressed, as observed in COVID 19 patients [48,62]. Our data show that IRF3 levels become undetectable in infected cells after 48 h; it is possible that effects driven by ISG15 ensure that IRF3 signalling is also repressed at earlier time points.

TAB1 is part of the TAB1/2/3/TAK1 complex [63] that regulates the activity of TAK1 (TGFβ-activated kinase 1), in response to different stimuli including TGFβ, IL1, TNFα and upon viral and bacterial infections [64,65]. TAK1 can then activate the NFκB pathway or signal through the MAP kinases pathway [66,67]. Lei *et al.* showed that the 3C protease of enterovirus 71 (EV-A71) cleaved TAB1 (along with TAB2, TAB3 and TAK1) at two cleavage sites (Q^414^S and Q^451^S) to perturb the formation of the complex and inhibit cytokine release downstream of NFκB [58]. Our identified cleavage site, at Q^444^S, forms a protein that is reminiscent of the second isoform of TAB1 (TAB1β, which lacks the C-terminus) that loses its interaction with TAK1 [68,69]. Further, the poly-Ser region (452-457) is a substrate for p38 kinase [70] (whose binding site has been mapped to residues 408–414 on TAB1) and phosphorylation controls cellular localization and activity of the protein [63]. Therefore, the loss of TAB1 C-terminus through 3CLpro cleavage would profoundly impact its ability to activate TAK1 and result in decrease production of cytokine through NFκB signalling.

Finally, we identified NLRP12 as a substrate of 3CLpro, and two cleavage sites could be identified. NLRP12, like other members of the NLRP family, possess a PYD domain, that binds the effector ASC through homotypic interaction, followed by a NACHT domain, which binds ATP and mediates activation of the protein, and a series of LR repeats that gives specificity to each member by modulating protein–protein interactions [71]. NLRP12, by similarity with NLRP3, is thought to be normally maintained in a monomeric, auto-inhibited conformation and release of this auto-inhibition is mediated by binding of a ligand (e.g. ATP) to the NACHT domain [72]. ATP binding to NLRP12 plays a major role in regulating the protein’s activity as it has been shown to not only induce self-oligomerisation, but also promote interaction with NFκB-induced kinase (NIK) and subsequent degradation of NIK [73]. Mutations in the ATP binding site are sufficient to increase production of proinflammatory cytokines and chemokines, mimicking loss of NLRP12 [73]. The first cleavage site in NLRP12, at residue 238, is located the NACHT domain, in between the two walker motifs that mediate ATP binding (walker A; residues 217–224 and walker B: residues 288-299, by analogy with NLRP3 [74]). This cleavage breaks the nucleotide binding site and whether this leads to activation or repression of the protein activity is an open question. Of note, this proteolytic cleavage also releases the PYD. PYD of ASC, the NLRP3/12 adaptor, has been shown to drive polymerization of ASC and formation of ASC specks upon inflammasome formation [75]. Whether NRLP12 PYD is able to polymerize on its own and induce downstream signalling and pyroptosis remains to be explored. The second cleavage site, at residue 938, releases 3 LRR motifs, and probably modifies protein–protein interactions.

Most relevant to COVID-19, NLRP12 is known to negatively regulate the release of pro-inflammatory cytokines [76] and pyroptosis has recently be identified as one possible explanation for the cytokine storm observed in severe cases of COVID-19 [77]. Mutations in NLRP12 [78] have been linked to autoinflammatory disorders, in particular the familial cold autoinflammatory syndrome 2 (FCAS2, OMIM: 611762). Of note, truncation R284X, close to the identified cleavage site has been identified in patient suffering from hereditary periodic fever syndrome and leads *in vitro* to a dramatic increase in NF-κB activation [79]. Up-regulation (and potentially over-activation) of NLRP12 has also been noted in patients with Kawasaki disease [80], a rare auto-inflammation of blood vessels in children. The emergence of Kawasaki-like syndromes in children positive for SARS-CoV-2 [81,82] could point to a molecular link between NLRP12 cleavage and inflammatory sur-activation.

### Comparison between species can facilitate interpretation of animal models for disease upon SARS-CoV-2 infection

The comparison of cleavage sites presented in Figure 6 once again highlights the difficulty of finding an animal model suitable for the study of SARS-CoV-2 infectivity and disease. In rodents, most of the typical species used for testing in laboratories (rat, hamsters, and mouse) have a mutation in the most important residue for PLpro cleavage. It has been rapidly established that mouse models of COVID-19 were ill-adapted as murine ACE2 the receptor for SARS-CoV-2, is significantly different to the human isoform [83]. This led to efforts in developing transgenic mice models with humanized ACE2 which was successfully used to study infection by SARS-CoV and SARS-CoV-2. According to our analysis however, this model may not fully recapitulate the lack of interferon production if this is driven by IRF3 cleavage. We have also shown that mouse NLRP12 is only cleaved once, compared to two cleavage sites for human NLRP12 (Figure 4(D,E)), although the functional impact of these cleavages remains to be studied. Ferrets are also a preferred model of respiratory viral infections and have been shown to be infected by SARS-CoV-2, but develop only mild clinical signs. So far, the most promising disease models amongst primates are rhesus macaques (*Macaca mulatta*) that develop pneumonia, although results of infection in capuchins (*Sapaju appella*) are yet to be published. In non-primates, cats present the most clinical signs with massive lung lesions [84].

### Analysis of PLpro and 3CLpro cleavage in bats and other potential host species

**Figure 8.**
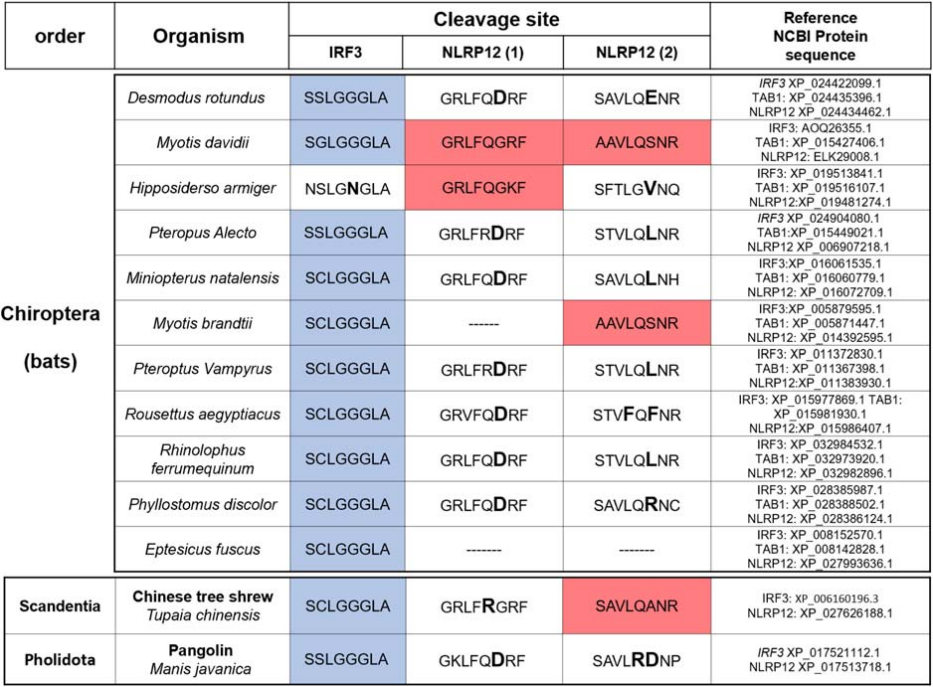
Analysis of cleavage sites in potential host species. Most “exotic” species that would be relevant for SARS-CoV, MERS or SARS-CoV-2 present the correct cleavage site for IRF3. This (limited) analysis identified only one species (*Myotis davidii*) that possess all 5 similar cleavage sites compared to human.

The fact that the two proteases of SARS-CoV-2 could have evolved to interfere with innate immunity is attractive. If one considers that most of evolution is driven by lucky side-effects, the additional targets IRF3, TAB1 and NLRP12 could give a selective advantage if their cleavage would either enhance transmission or delay the response to infection. We extended our comparison of cleavage sites to include wild animals that could have been host reservoirs of the virus, hypothesizing that the viral proteases had evolved to target innate immune proteins of their host. To date, the exact reservoir and intermediate hosts of the virus remain to be found. It is highly probable that the virus originates from a bat coronavirus and the Malayan pangolins (*Manis javanica*) might have acted as intermediate hosts. To this end, we compared the IRF3 and NLRP12 sequences in different bats, the Malayan pangolin and the Chinese tree shrew (*Tupaia chinensis)*, based on data availability (Figure 8). Data for the masked palm civet (*Paguma larvata*) are not available; this is unfortunate as this species was determined to be the intermediate host for SARS-CoV.

As before, the IRF3 sequence around the cleavage site was remarkably conserved amongst species. On the contrary, the sequences for NLRP12 are highly divergent, especially at the C-terminus. Interestingly, we found that one species of bats, Davids’ myotis, presents the three cleavage sites in IRF3 and NLRP12 (and also the two cleavage sites in TAB1). The presence of the five human-like cleavage sites for IRF3, TAB1 and NLRP12 in a single species shows that it is possible that the SARS viruses could have gained the new functionality of cleaving these Human Innate Immune Proteins in a single reservoir host, potentially in *Myotis Davidii*. The fact that *Myotis davidii* can be found near the epicentre of the SARS-CoV-2 pandemic makes it a possible candidate for a previous reservoir host, even if it does not exclude other hosts for SARS-CoV and SARS-CoV-2. Another small animal found in the same province, the Chinese tree shrew, displays at least two cleavage sites for IRF3 and NLRP12; the first cleavage site in NLRP12 (KLFRG) may potentially be cleaved. The species of pangolin described in China (*Manis Javanica*) does not possess the NLRP12 cleavage sites (note that surprisingly, an African pangolin presents all three cleavage sites identical to humans). Both cleavage sites of TAB1 are exactly conserved across all these species.

The current pandemic is so large that the possibility of spillback has been raised [85]: due to the number of infected humans, one can imagine that wild animals would be infected by contact with humans, and constitute new reservoirs of the virus. With this in mind, it would be interesting to see whether *Myotis davidii* would be more susceptible to SARS-CoV-2, and if IRF3 and NLRP12 levels are affected during infection, or if the unique features implicated in chiopteran tolerance for multiple viruses would still protect this species.

## Conclusion

Overall, the method presented here enables medium to high-throughput screen of the activity of viral protein or bacterial effectors and will help design new antiviral and antibiotic strategies. In this study, we presented the results on the first 71 HIIPs (Human Innate Immune Proteins), and the screen will be expanded to cover more potential targets and pathways in the future. Our results show that in addition to the de-ubiquitinase activity of nsp3, SARS-CoV-2 uses its two proteases to further impact the host innate immune signalling. Our results were validated in SARS-CoV-2 infected A293T-Ace2 cells, where decrease of IRF3, NLRP12 and TAB1 was demonstrated by Western Blotting. Our findings of IRF3 cleavage are consistent with the literature and the previous work showing an inhibition of interferon beta production and immunodepression at the early stages of SARS-CoV-2 infection. More importantly, the direct cleavage of NLRP12 by 3CLpro could explain the hyper-inflammation observed in severe cases of COVID-19, potentially by pyroptosis-induced cytokine storm [77].

## Supplementary Material

IRF3_NLRP12_TAB1_-_Supplementary_Information_2_editable.doc
