# Supplementary material for: SARS-CoV-2 proteases PLpro and 3CLpro cleave IRF3 and critical modulators of inflammatory pathways (NLRP12 and TAB1): implications for disease presentation across species
Source: Emerg Microbes Infect. 2021 Jan 29;10(1):178–95. doi: 10.1080/22221751.2020.1870414 (PMC7850364; doi:10.1080/22221751.2020.1870414)
Supplement: IRF3_NLRP12_TAB1_-_Supplementary_Information_2_editable.doc [file TEMI_A_1870414_SM0238.doc]

1

2

3

4

5

6

7

8

SUPPLEMENTARY INFORMATION

**SARS-CoV-2 proteases cleave IRF3 and critical modulators of inflammatory pathways (NLRP12 and TAB1): implications for disease presentation across species.**

1. Mehdi Moustaqil*^,1^, Emma Ollivier*^,1^, Hsin-Ping Chiu^2^, Sarah Van Tol^3^, Paulina Rudolffi-Soto^1^, Christian
2. Stevens^2^, Akshay Bhumkar^1^, Dominic J.B. Hunter^1,4^, Alex Freiberg^3^, David Jacques^1^, Benhur Lee^2,+^, Emma
3. Sierecki^1,+^, Yann Gambin^1,+^

12

13 ^1^ EMBL Australia Node for Single Molecule Sciences, and School of Medical Sciences, Botany Road, The University

1. of New South Wales, Sydney NSW 2052 Australia
2. ^2^ Department of Microbiology, Icahn School of Medicine at Mount Sinai, One Gustave L Levy Place #1124 New
3. York, NY 10029 USA
4. ^3^ Department of Pathology, Robert E. Shope BSL-4 Laboratory and Center for Biodefense & Emerging Infectious
5. Diseases, The University of Texas Medical Branch, 301 University Boulevard Galveston, TX 77555 USA
6. ^4^ Institute for Molecular Biosciences, The University of Queensland, 306 Carmody Road St Lucia Qld 4072 Australia
7. * these authors contributed equally

| 23 | + | correspondence should be addressed to [y.gambin@unsw.edu.au,](mailto:y.gambin@unsw.edu.au) | [e.sierecki@unsw.edu.au](mailto:e.sierecki@unsw.edu.au) and |
| --- | --- | --- | --- |
|  |

1. benhur.lee@mssm.edu

25

26

1

1. **Supplementary Figure 1:**


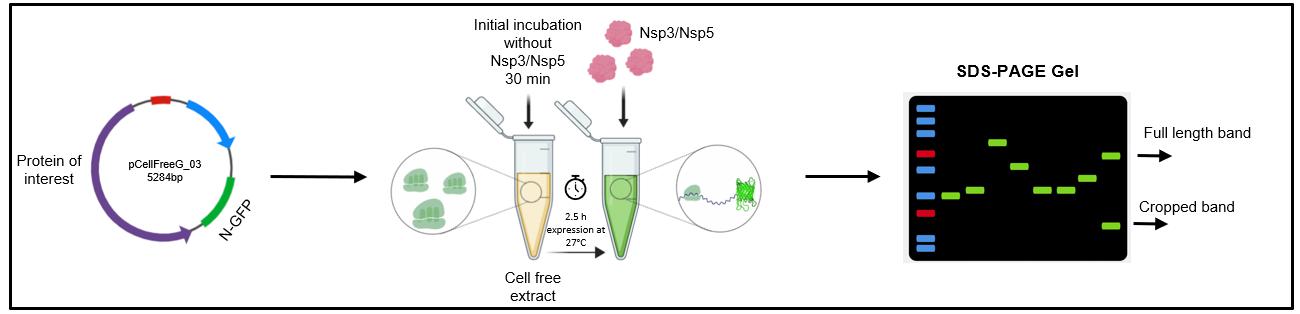


28

1. **Supplementary Figure 1: screen of proteolytic cleavage of Human Innate Immune Proteins (HIIPs)**
2. Open Reading Frames (ORFs) encoding 71 human innate immune proteins were cloned into Gateway vectors
3. for cell-free expression as GFP-fusions. After mixing with the PLpro or 3CLpro proteases of SARS-CoV-2,
4. the proteins were expressed for 2 ½ hours and analysed by SDS-page gels. The cleavage of the HIIPS creates
5. additional fluorescent bands in the migration of the GFP-tagged proteins.
6. **Supplementary Figure 2**


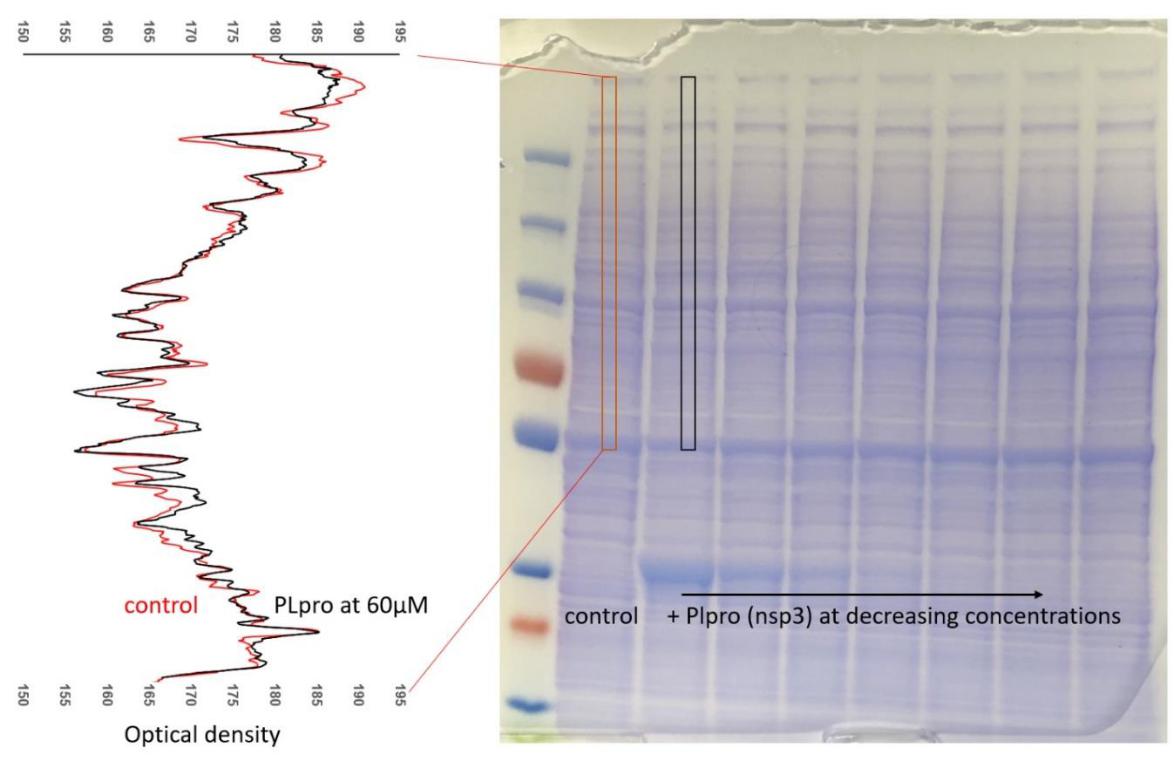


35

1. **Supplementary Figure 2: controlling for non-specific cleavage of proteins from LTE expression system.**
2. The cell-free expression system was loaded onto the SDS-page gels following the classic protocol and stained
3. by Coomassie to reveal the proteins present in the LTE system. The SARS-CoV-2 protease PLpro (nsp3) was
4. added at concentration ranging from 60 µM to 1µM (same as in Figure 2A). The gel shows on the right that
5. the banding pattern of LTE is not affected by PLpro. (left): the density of bands across the control (LTE without
6. PLpro added) was compared to the one obtained when 60µM of purified PLpro was added in the expression
7. system. No significant differences were noted, confirming that PLpro doesn’t have a non-specific cleavage
8. activity on the proteins of LTE. Also, the intensity of the GFP-tagged protein bands on the gels did not vary
9. significantly when PLpro or 3CLpro were added, suggesting that the expression levels were unaffected, and
10. that the components of the cell-free systems essential for expression were not cleaved.

2

46

1. **Supplementary Figure 3:**

48


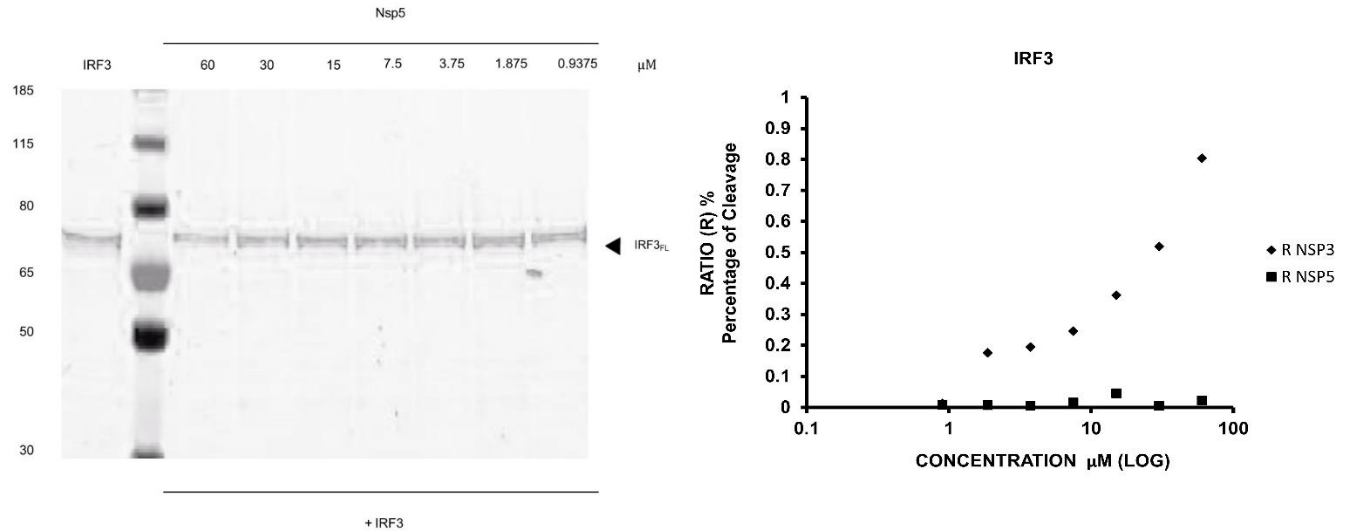


49

1. **Supplementary Figure 3: quantification of IRF3 cleavage by NSP3 and NSP5**
2. (left): SDS-page analysis of the cleavage of IRF3 protein, with a N-terminal GFP tag. The protein was
3. expressed alone or in the presence of increasing concentrations of the SARS-CoV-2 protease3CLpro (nsp5).
4. The gel shows no cleavage site. (right): Dose-response curve of SARS-CoV-2 protease PLpro (nsp3) and
5. 3CLpro (nsp5) on Human IRF3. The result from the SDS-page gel was processed using IMAGE J and the
6. Percentage of cleavage (Ratio R) was calculated by measuring the length of the line, in pixels.

58

1. **Supplementary Figure 4:**

60


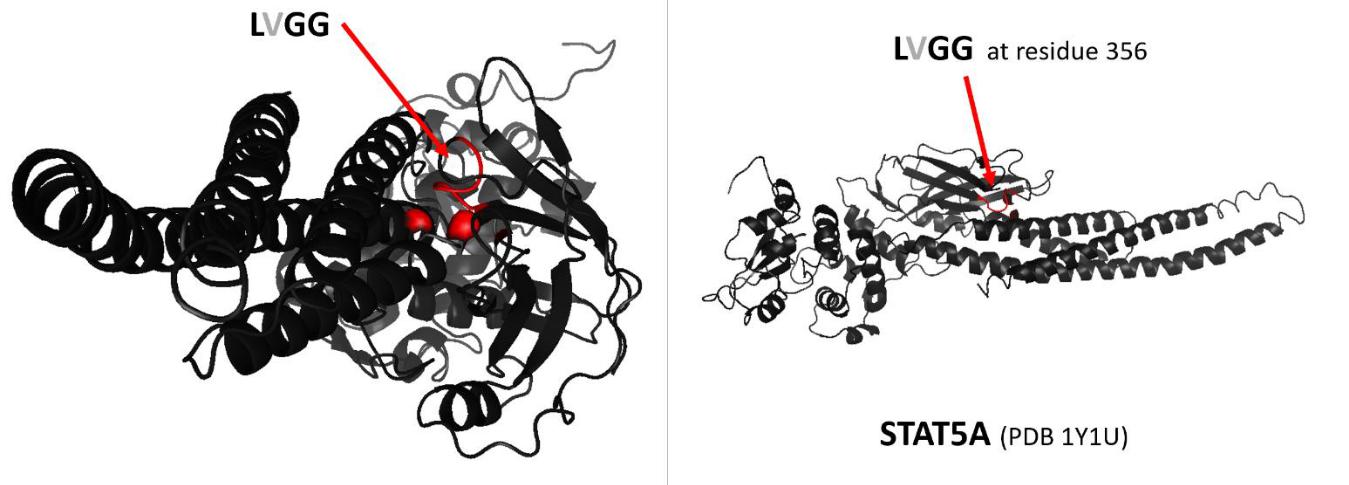


1. **Supplementary Figure 4: localization of a PLpro putative cleavage sequence in STAT5A**
2. In our experiments, the protein STAT5A is not cleaved by PLpro or 3CLpro of SARS-CoV-2

63

64

3

1. **Supplementary Figure 5:**


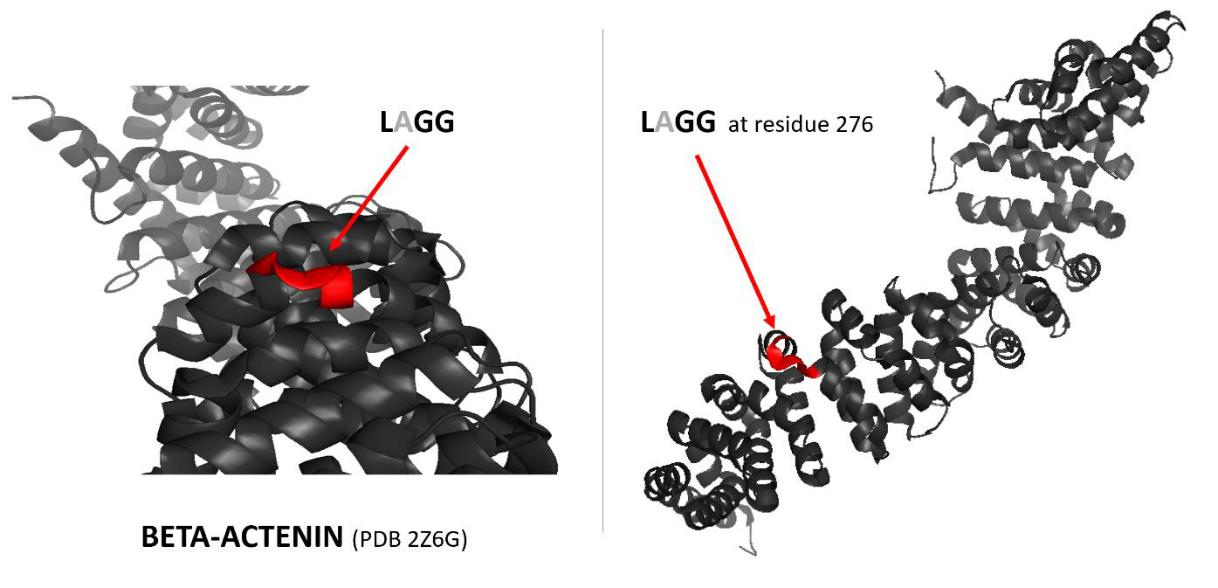


66

1. **Supplementary Figure 5: localization of a PLpro putative cleavage sequence in beta-Catenin**
2. In our experiments, the protein beta-Catenin is not cleaved by PLpro or 3CLpro of SARS-CoV-2

69

1. **Supplementary Figure 6:**


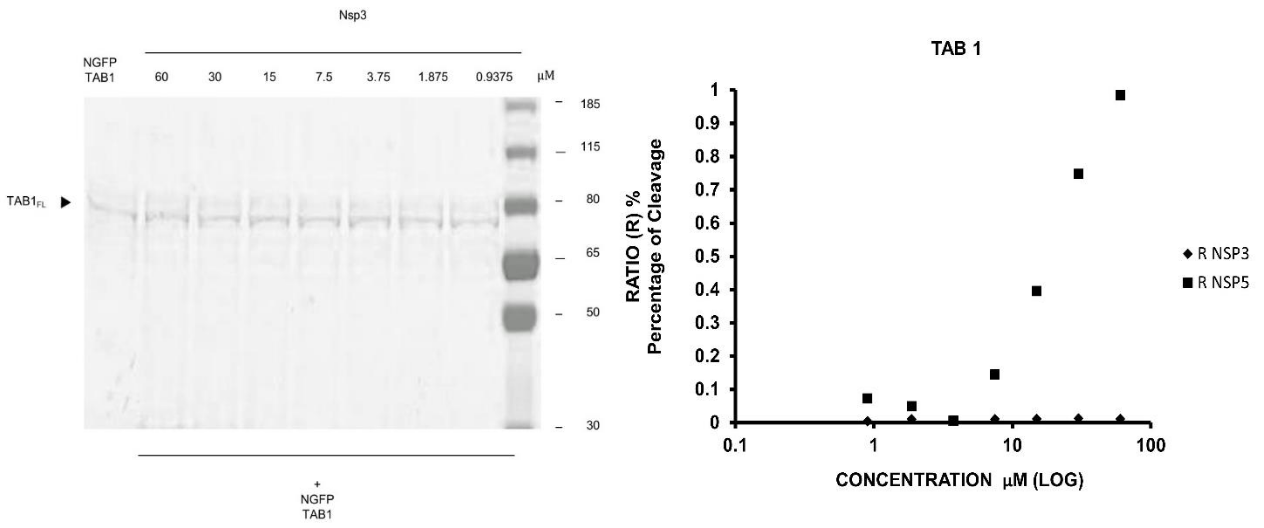


71

1. **Supplementary Figure 6: quantification of TAB1 cleavage with 3CLpro and PLpro**
2. (left): SDS-page analysis of the cleavage of TAB1 protein, with a N-terminal GFP tag. The protein was
3. expressed alone or in the presence of increasing concentrations of the SARS-CoV-2 protease PLpro (nsp3).
4. The gel shows no cleavage site. (right): Dose-response curve of SARS-CoV-2 protease PLpro (nsp3) and
5. 3CLpro (nsp5) on Human TAB1. The result from the SDS-page gel was processed using IMAGE J and the
6. Percentage of cleavage (Ratio R) was calculated by measuring the length of the line, in pixels.

78

4

1. **Supplementary Figure 7:**


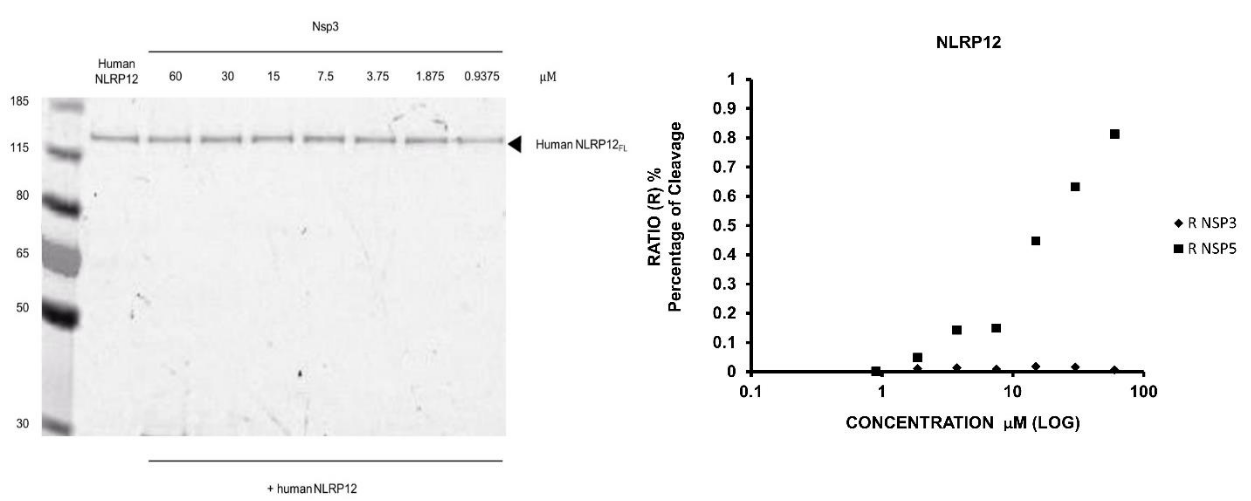


80

1. **Supplementary Figure 7: quantification of NLRP12 cleavage with 3CLpro and PLpro**
2. (left): SDS-page analysis of the cleavage of NLRP12 protein, with a N-terminal Cherry tag. The protein was
3. expressed alone or in the presence of increasing concentrations of the SARS-CoV-2 protease PLpro (nsp3).
4. The gel shows no cleavage site. (right): Dose-response curve of SARS-CoV-2 protease PLpro (nsp3) and
5. 3CLpro (nsp5) on Human NLRP12. The result from the SDS-page gel was processed using IMAGE J software
6. and the Percentage of cleavage (Ratio R) was calculated by measuring the length of the line, in pixels.

87

1. **Supplementary Figure 8:**


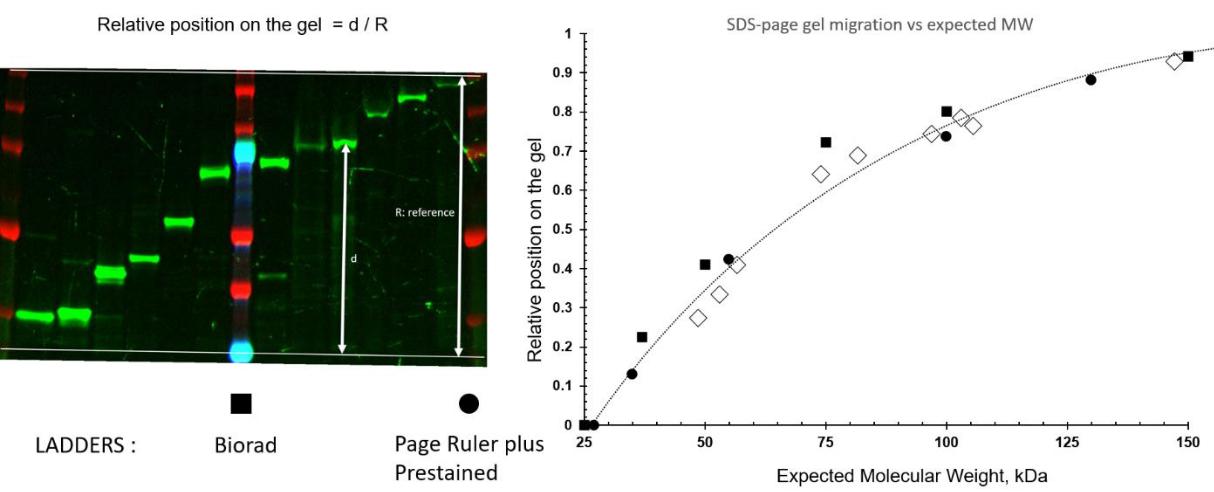


89

1. **Supplementary Figure 8: calibration of the migration of GFP-tagged proteins on SDS-page**
2. **(left):** a range of HIIPs were selected with different molecular weights, ranging from 15 to 140 kDa; the HIIPS
3. were expressed in LTE, mixed with LDS after 3h of expression, and separated on the SDS-page gel. In this
4. gel, two different ladders were used to calibrate migration of 12 proteins of interest. The Page ruler Prestained
5. ladder has been developed specifically for the 4-12% Bis-Tris gels (ThermoFisher, black ●) and seems more
6. accurate to predict sizes of GFP-tagged proteins (in white diamonds ◊). The trendline indicated on the graph
7. (right) will be used to estimate the size of the GFP-tagged fragments of TAB1 and NLRP12.

97

5

1. **Supplementary Figure 9:**

99


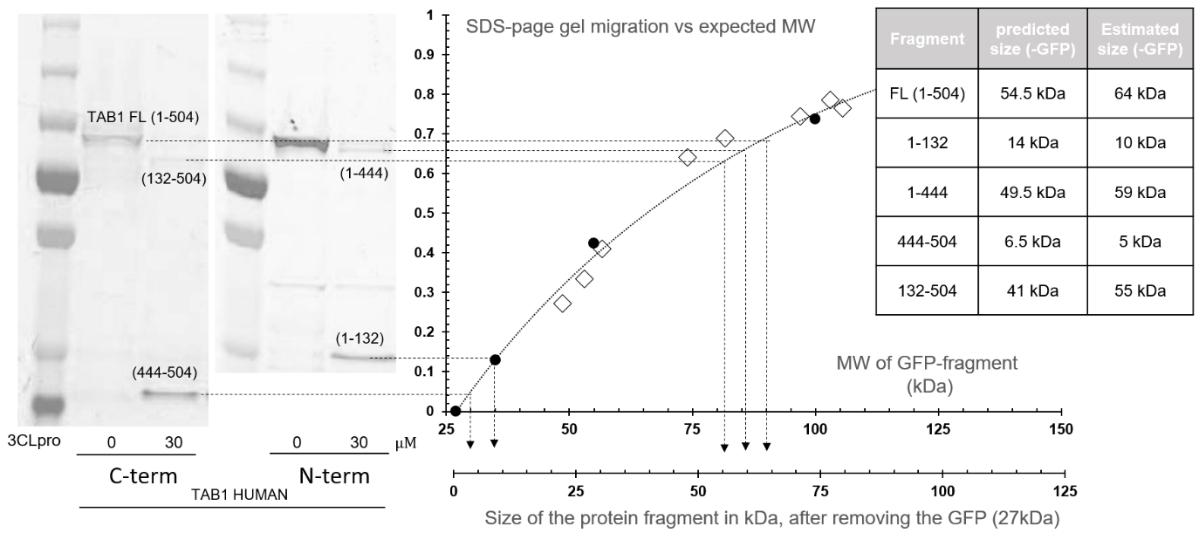


1. **Supplementary Figure 9: analysis of TAB1 fragments upon 3CLpro cleavage**
2. Probably due to the non-denaturation of the protein TAB1 in our LDS loading protocol, the full-length protein
3. human TAB1 migrates slower than expected on the SDS-page gel (estimated size 64 kDa from the calibration,
4. vs 54.4 kDa expected). Nevertheless, the variations in size due to 3CLpro cleavage are consistent with the two
5. sites at position 132 and 444. On the gel, the fragments are indicated, and the migration was analysed on the
6. master curve to estimate the size of the GFP-tagged fragments. The 27kDa of the GFP were taken into account
7. in the table (right) to compare the predicted and observed sizes of the proteins and protein fragments.

107

6

1. **Supplementary Figure 10:**

109
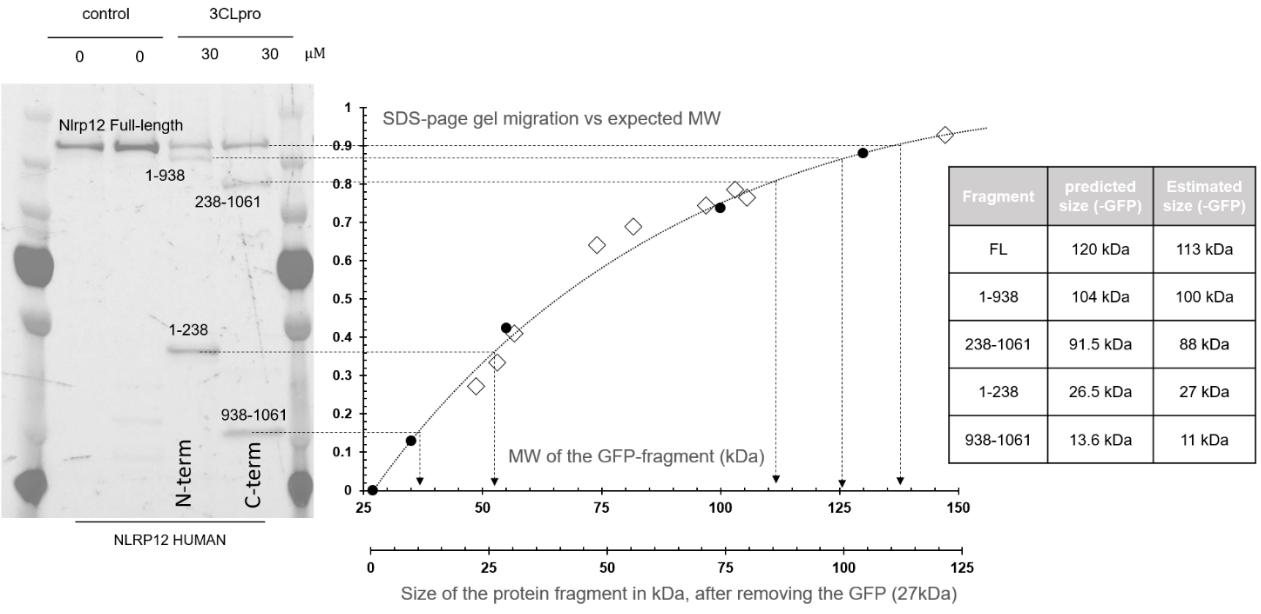


1. **Supplementary Figure 10: analysis of NLRP12 fragments upon 3CLpro cleavage**
2. The full-length human NLRP12 proteins, tagged in N-term and C-term with GFP, were mixed with 3CLpro
3. and analysed by SDS-page gels. The five bands obtained for the full-length NLRP12 and the four fluorescent
4. cleavage products were analysed on the predictive size/migration plot. The variations in size are perfectly
5. consistent with the two cleavages sites at position 238 and 938. On the SDS-page gel on the left, the fragments
6. are indicated, and the migration was analysed on the master curve to estimate the size of the GFP-tagged
7. fragments. The sizes obtained (after removing the 27kDa contribution of the GFP) were compared to the
8. predicted sizes of the proteins and protein fragments.

118

7

1. **Supplementary Figure 11:**

120


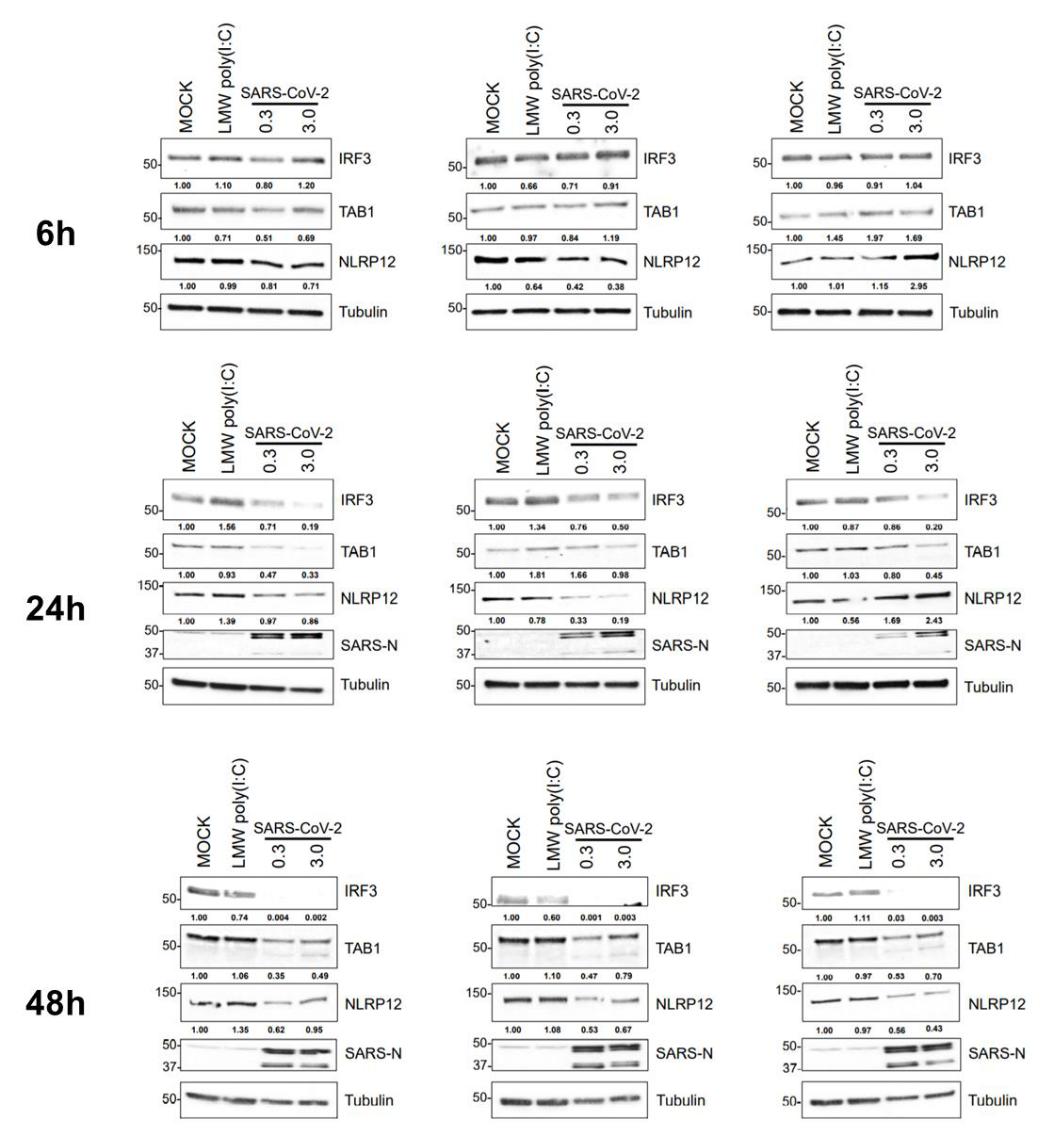


121

122

1. **Supplementary Figure 11: analysis of IRF3, TAB1 and NLRP12 levels in SARS-CoV-2 infected cells.**
2. These Western Blots correspond to the triplicate experiments described in Figure 5. The experiments were
3. conducted in the Biosafety Level 4 facility of the Galveston National Laboratory at the University of Texas
4. Medical Branch (UTMB, Texas, USA).

127

8

1. **Supplementary Figure 12:**

129


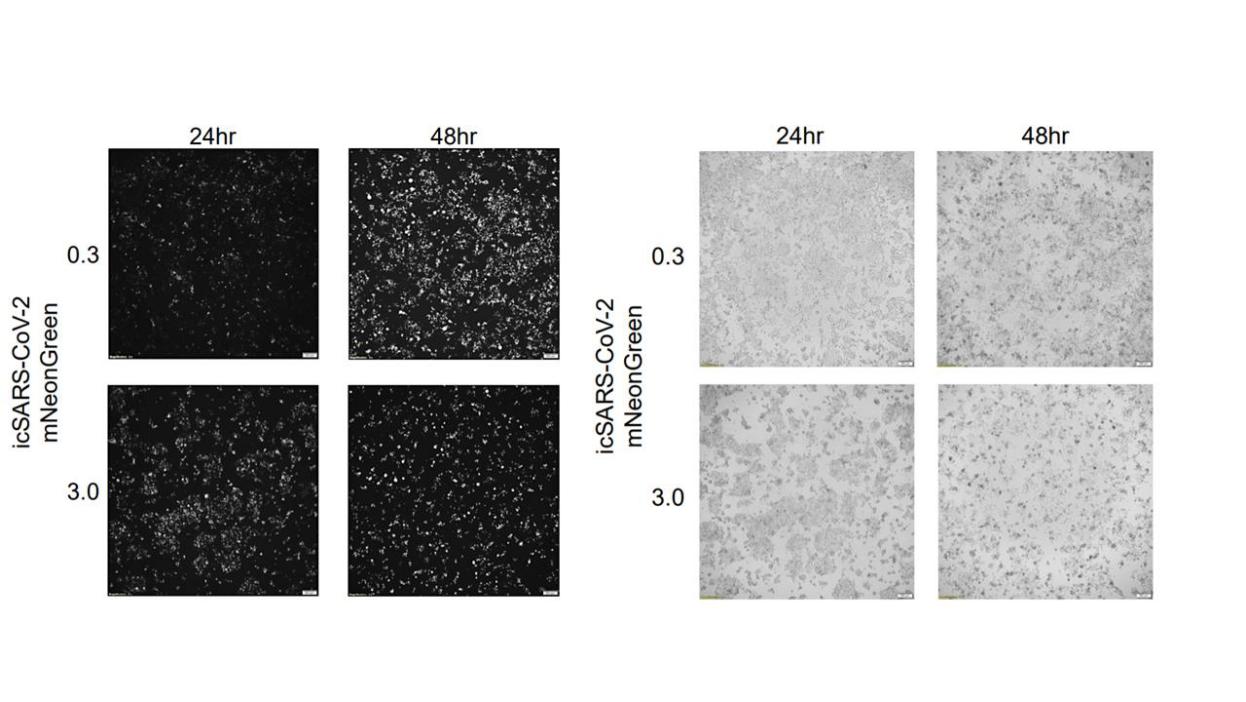


1. **Supplementary Figure 12: imaging of icSARS-CoV-2 mNeonGreen infected cells,** using mNeonGreen
2. fluorescence (left) and brightfield (right), at two different MOI (0.3 and 3.0), at 24h and 48h.

132

1. **Supplementary Figure 13:**


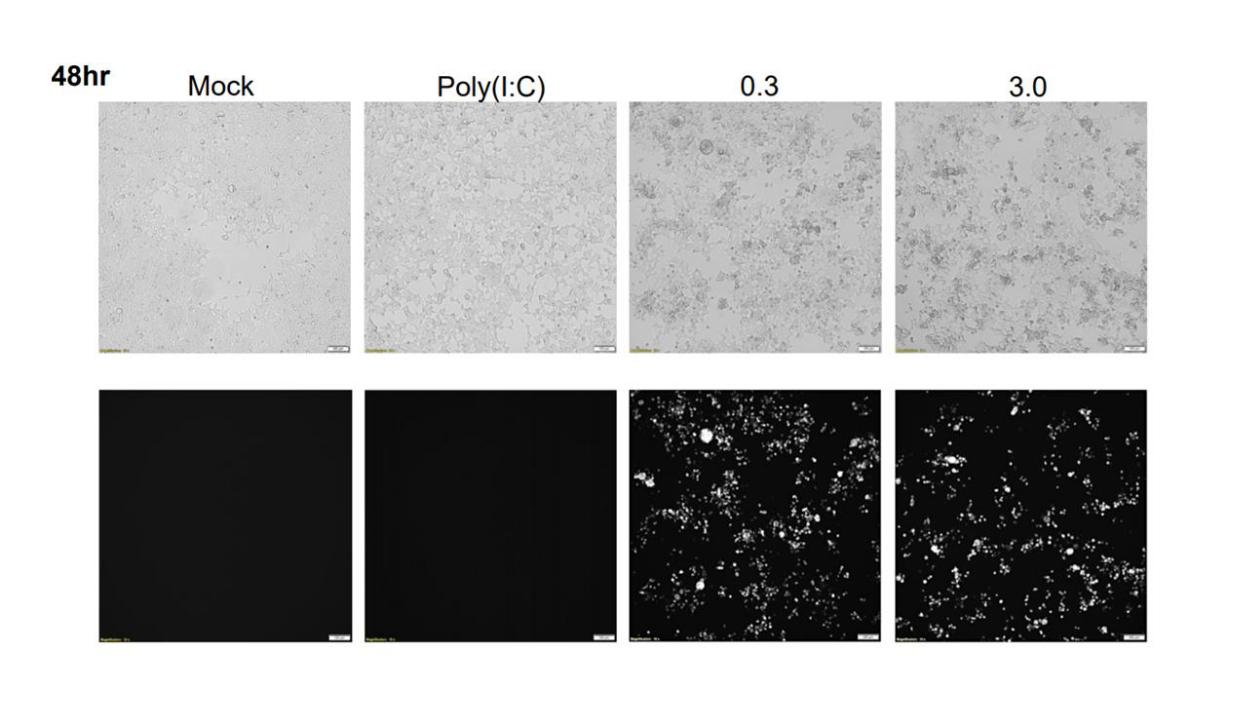


134

1. **Supplementary Figure 13: imaging of icSARS-CoV-2 mNeonGreen infected cells,** using brightfield (top)
2. and mNeonGreen fluorescence (bottom) at 48h post-infection, for Mock conditions, Poly(I:C) and the two
3. different MOI (0.3 and 3.0).

138

9

1. **Supplementary Figure 14:**


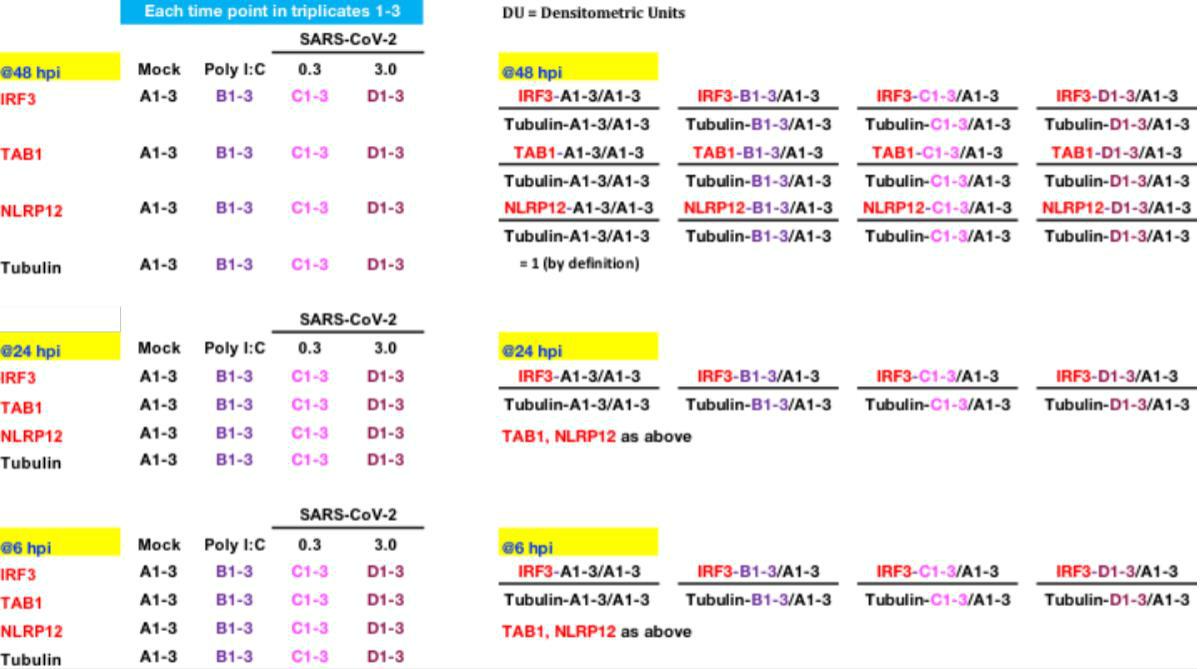


140

141

1. **Supplementary Figure 14: normalization of the data presented in Figure 5 (D, E F).**
2. To account for possible cytopathic effect, the expression levels of the IRF3, TAB1 and NLRP12 proteins were
3. normalized by the amount of tubulin for each condition. All protein bands measured by densitometry were
4. normalized to the relative amount of tubulin under that experimental condition, at each given time point. Then,
5. the levels of IRF3, TAB1 and NLRP12 bands in the various experimental conditions (SARS-CoV-2 infection
6. at MOI 3.0 and MOI 0.3 plus PolyI:C) were compared to the cognate Mock control.

148

10
